# Cancer-associated fibroblast-derived exosome microRNA-21 promotes angiogenesis in multiple myeloma

**DOI:** 10.1038/s41598-023-36092-6

**Published:** 2023-06-14

**Authors:** Sun Miaomiao, Wang Xiaoqian, Shou Yuwei, Chen Chao, Yang Chenbo, Liang Yinghao, Hong Yichen, Shu Jiao, Chen Kuisheng

**Affiliations:** 1 Department of Pathology, The First Affiliated Hospital of Zhengzhou University, Zhengzhou, People’s Republic of China; 2 BGI College, Zhengzhou University, Zhengzhou, People’s Republic of China; 3 Henan Province Key Laboratory of Tumor Pathology, Department of Pathology, The First Affiliated Hospital of Zhengzhou University, Zhengzhou, People’s Republic of China

**Keywords:** Myeloma, Tumour angiogenesis, Tumour biomarkers

## Abstract

Multiple myeloma (MM) is the second most common hematological malignancy, and angiogenesis determines its progression. In the tumor microenvironment, normal fibroblasts (NFs) are transformed into cancer-associated fibroblasts (CAFs), which can promote angiogenesis. Microribonucleic acid-21 (miR-21) is highly expressed in various tumors. However, research on the relationship between tumor angiogenesis and miR-21 is rare. We analyzed the relationship between miR-21, CAFs, and angiogenesis in MM. NFs and CAFs were isolated from the bone marrow fluids of patients with dystrophic anemia and newly-diagnosed MM. Co-culturing of CAF exosomes with multiple myeloma endothelial cells (MMECs) showed that CAF exosomes were able to enter MMECs in a time-dependent manner and initiate angiogenesis by promoting proliferation, migration, and tubulogenesis. We found that miR-21 was abundant in CAF exosomes, entering MMECs and regulating angiogenesis in MM. By transfecting NFs with mimic NC, miR-21 mimic, inhibitor NC, and miR-21 inhibitor, we found that miR-21 significantly increased the expression of alpha-smooth muscle actin and fibroblast activation protein in NFs. Our results showed that miR-21 can transform NFs into CAFs, and that CAF exosomes promote angiogenesis by carrying miR-21 into MMECs. Therefore, CAF-derived exosomal miR-21 may serve as a novel diagnostic biomarker and therapeutic target for MM.

## Introduction

Multiple myeloma (MM), caused by the abnormal proliferation of plasma cells and overproduction of monoclonal immunoglobulin in the bone marrow, is the second most common hematological malignancy^1–3^. According to the Global Cancer Report, 117,077 people died of MM in 2020, accounting for 1.2% of all cancer-related deaths^4,5^. The median survival rate of patients with MM has been extended by 8–10 years by the introduction of multiple treatments; however, it remains incurable. MM is a vascular-rich tumor, and angiogenesis is involved in its progression, which therefore determines its treatment and prognosis^6^. Recently, an increasing number of studies have shown that the various components of the tumor microenvironment are involved in MM tumor angiogenesis^7,8^.

Cancer-associated fibroblasts (CAFs) constitute a heterogeneous population in the tumor microenvironment^9^. Fibroblast activation protein (FAP) and alpha-smooth muscle actin (α-SMA) are highly expressed in CAFs and can be used to identify them^10,11^. Normal fibroblasts (NFs) are the main source of CAFs^12,13^. NFs can be activated to become CAFs by cytokines derived from tumor or other mesenchymal cells, as well as by oncoproteins such as c-Ski^14^. Recent studies have found that dysregulation of non-coding ribonucleic acid (ncRNA) such as microRNA-21 (miR-21), microRNA-199a (miR-199a), and micoRNA-214 (miR-214) are involved in CAF formation^15–17^. CAFs regulate the biological behaviors of tumors through various mechanisms. Angiogenesis is an important function of CAFs in the regulation and development of tumorigenesis^18,19^. Studies have shown that miRNAs derived from CAFs are closely related to their angiogenesis-promoting effects^20,21^. Abnormally-expressed miRNAs can regulate tumor angiogenesis by acting on endothelial cells.

Exosomes exist in the tumor microenvironment and have long half-lives, high stabilities, easy separation, and long-term persistence^22,23^. They can carry DNA, RNA, miRNA, and other substances from the source cell into the recipient cell and mediate the transfer of material information between cells^24^. The most widely studied nucleic acid substances in exosomes are miRNAs, which can promote tumor angiogenesis^25–28^. Studies have found that exosomal miRNAs produced by CAFs are involved in biological behavior regulation of tumors through cell-to-cell communication with miRNAs in tumor cell exosomes ^29^. The miR-21 is abnormally overexpressed in a variety of cancers, including MM, and is one of the earliest experimentally-validated proto-oncogenic miRNAs^30,31^. Studies have shown that miR-21 is closely related to the activation, proliferation, migration, apoptosis, and angiogenesis of vascular endothelial cells, and that it can increase the density and number of blood vessels in vivo^32^. Furthermore, some studies have found that overexpression of miR-21 in prostate cancer tissue increases the expression of HIF-1α and VEGF by regulating VHL, thereby inducing angiogenesis^33^. Other studies have shown that miR-21 expression may positively regulate HIF-1α by inhibiting VHL, further playing a role in promoting tumor angiogenesis by regulating the HGF/c-Met signaling pathway^34^. However, the relationship between cancer-associated fibroblast (CAF)-derived exosomal miR-21 and MM angiogenesis remains unclear.

In the earlier stage of our research on this subject, we used bioinformatics to screen human CAFs exosomal miRNAs (GSE77318) and CAFs miRNA (GSE184991) from GEO databases. We concurrently screened miRNAs related to angiogenesis (GSE50437) and took the intersection of miRNAs. We found miR-21 to be one of the miRNAs most closely related to angiogenesis in CAF exosomes. In this study, we isolated primary CAFs from the bone marrow fluids of patients with newly-diagnosed MM and demonstrated that exosomal miR-21 promoted MM angiogenesis by regulating the proliferation, migration, and tubular formation abilities of multiple myeloma endothelial cells (MMECs). In addition, we found that miR-21 activated NFs, turning them into CAFs.

## Results

### Establishment and identification of NFs and CAFs

**Figure 1:**
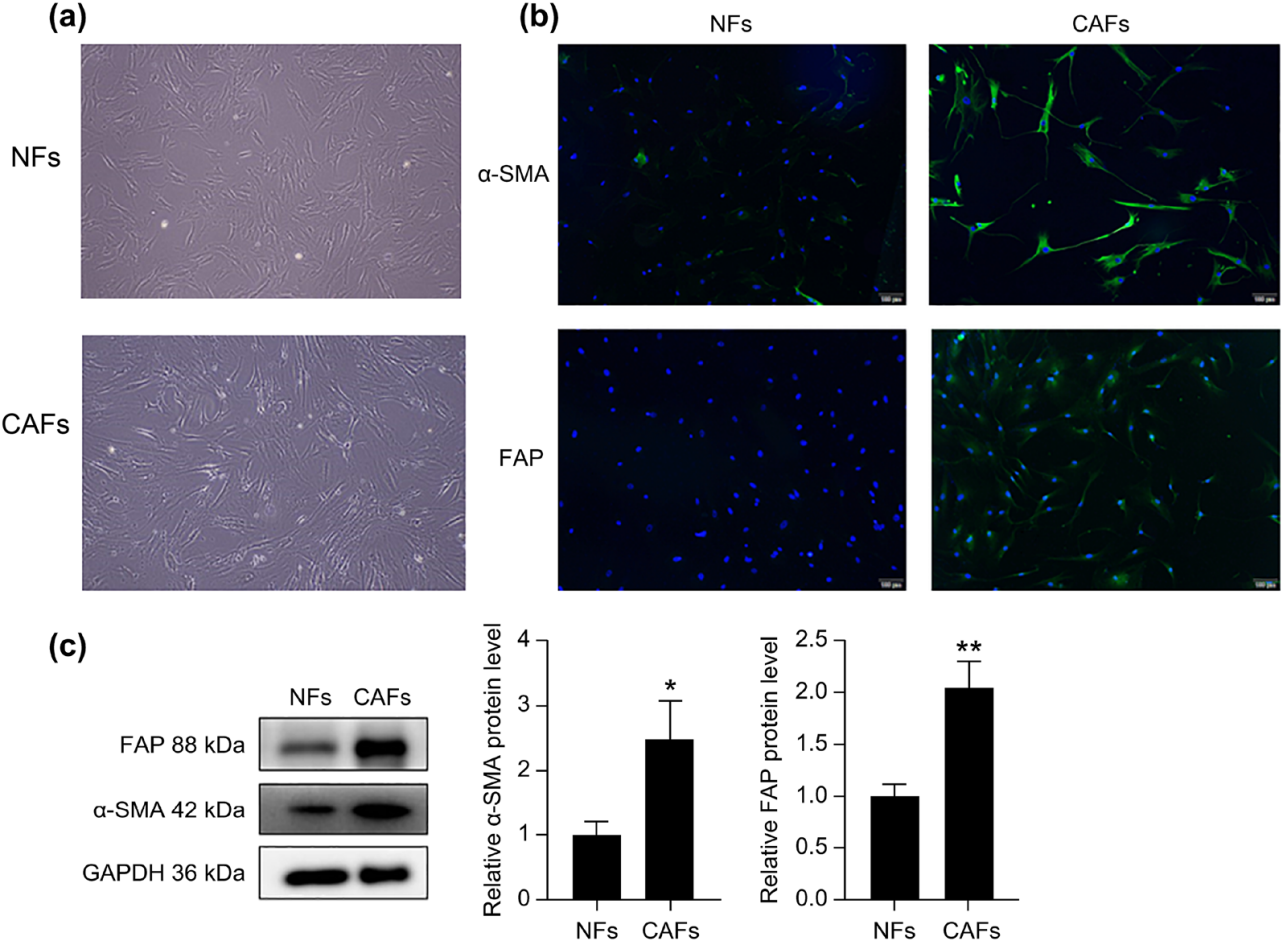
Isolation and identification of primary NFs and CAFs. (**a**) The morphologies of NFs from patients with dystrophic anemia and CAFs from patients with newly-diagnosed MM were observed by light microscopy (40 ×); (**b**) Expression levels of α-SMA and FAP in NFs and CAFs were detected by immunofluorescence and (**c**) Western blot. *p < 0.05; **p < 0.01.

NFs and CAFs were isolated and purified from the iliac bone marrow fluids of patients with dystrophia anemia who had been newly diagnosed with MM. Both the NFs and CAFs presented spindle-like morphologies of fibroblast ministerologies (Fig. 1a). To assess the purities and phenotypes of the isolated NFs and CAFs, we examined α-SMA and FAP expression. Immune cell fluorescence showed that the fluorescence intensities of the NFs were weak, and that α-SMA and FAP were barely expressed, whereas the fluorescence intensities of the CAFs were strong, with abundant expression of α-SMA and FAP (Fig. 1b). Western blotting (WB) results showed that α-SMA and FAP were expressed in both NFs and CAFs. Compared to NFs, α-SMA (*p* < 0.05) and FAP (*p* < 0.01) levels were significantly higher in CAFs (Fig. 1c). Based on these results, NFs and CAFs were successfully separated.

### Isolation and identification of exosomes

**Figure 2:**
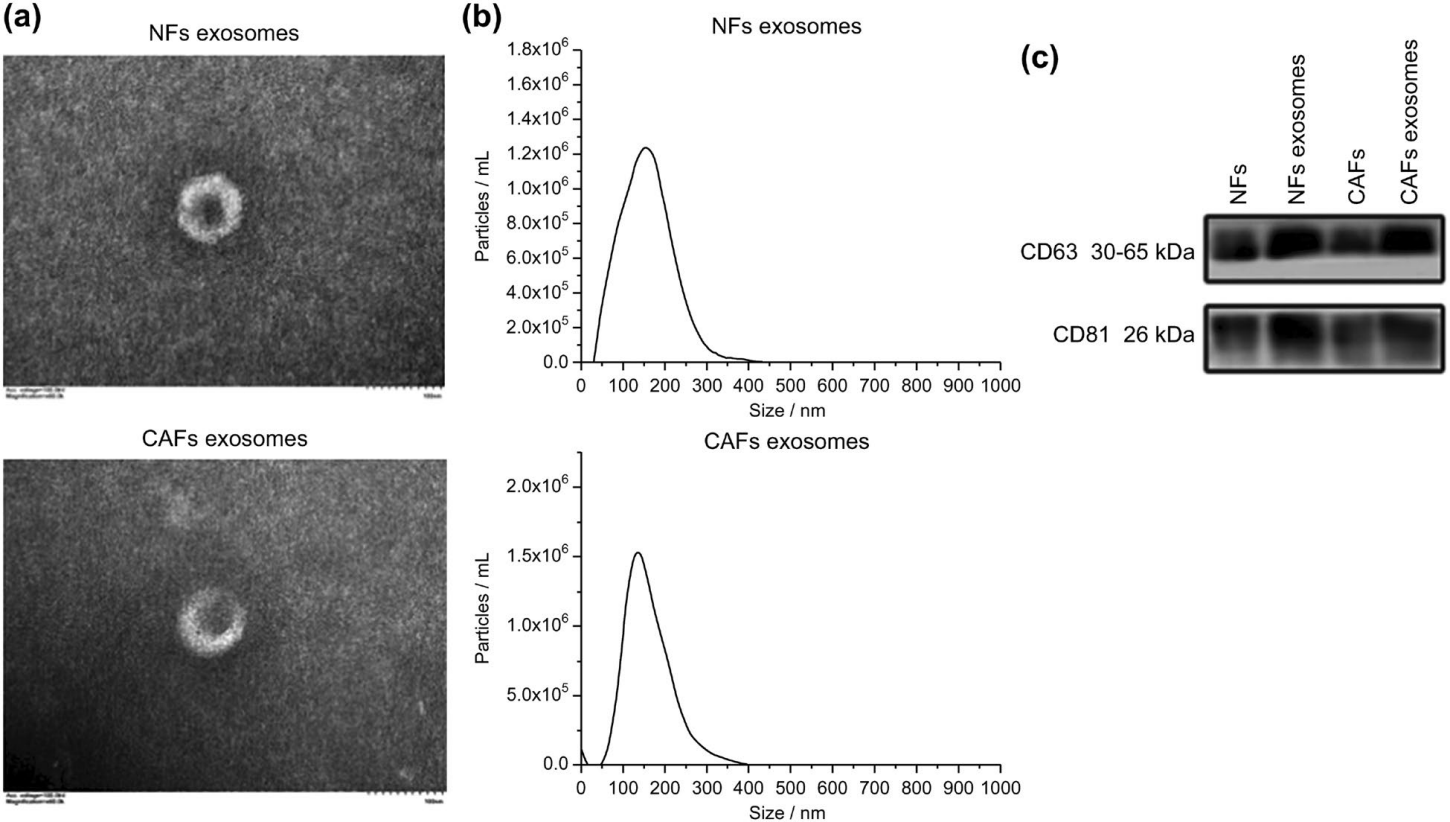
Extraction and identification of exosomes. (**a**) Transmission electron microscopy, (**b**) NTA, and (**c**) WB were used to observe the morphologies, particle size distributions, and the expression levels of CD63 and CD81 proteins in the cells and exosomes, respectively.

Exosomes were isolated from the supernatants of NFs and CAFs using an Exoquick-TC kit, and identified by transmission electron microscopy, WB, and nanoparticle tracking analysis (NTA). The results showed that the isolated exosomes were tea-saucer shaped, had a bilayer structure (Fig. 2a), and highly expressed both CD63 and CD81 (Fig. 2c). The particle sizes of the NF and CAF exosomes were approximately 138.8 nm, and 127.2 nm (Fig. 2b), respectively. This indicated that we had successfully isolated the exosomes.

### RPMI-8226 activation of human umbilical vein endothelial cells (HUVECs) into MMECs

**Figure 3:**
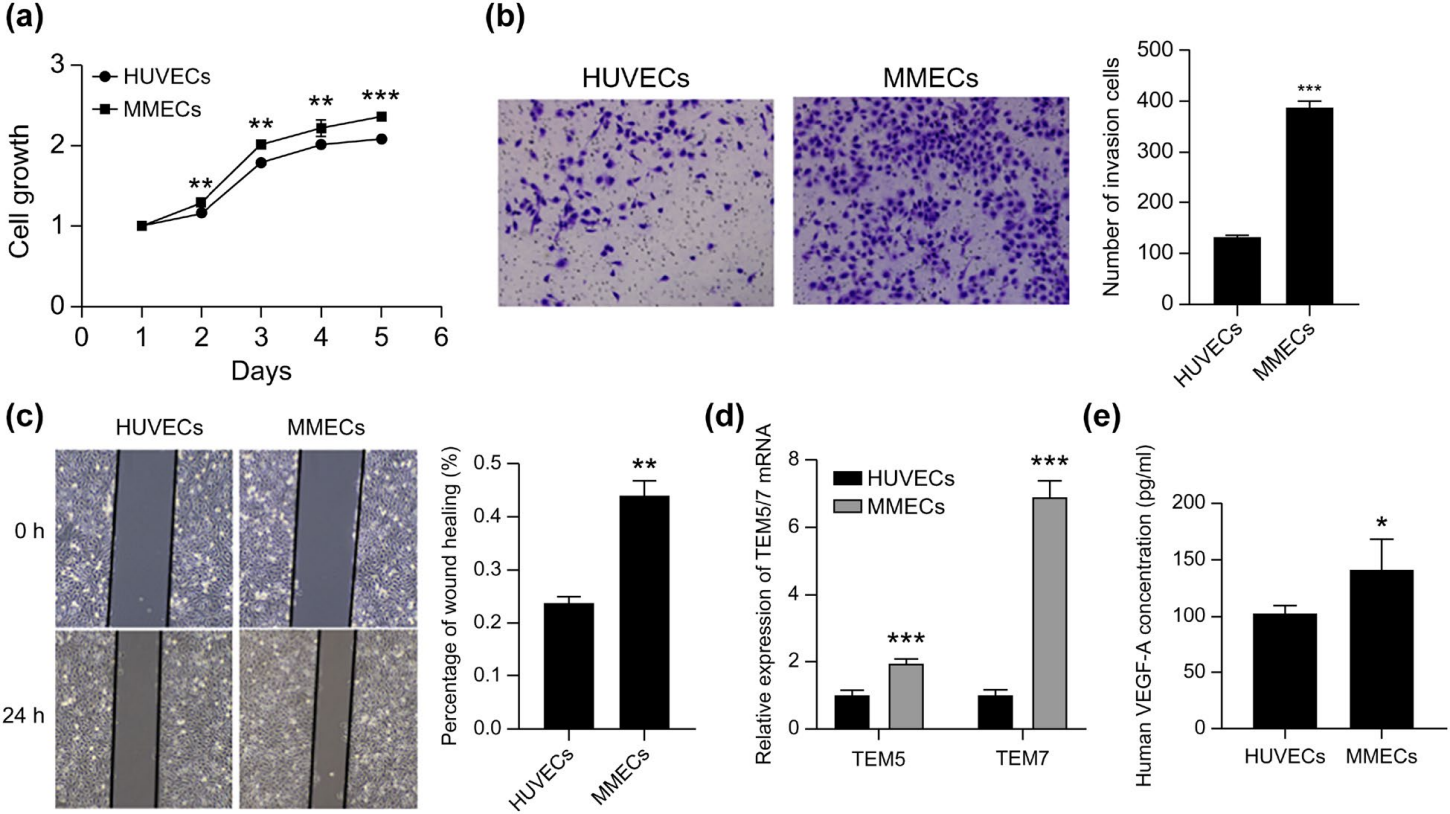
Induction and identification of myeloma-associated endothelial cells. (**a**) CCK-8, (**b**) a transwell invasion test, and (**c**) transwell migration test were used to detect the proliferation, invasion, and migration abilities of MMECs and HUVECs. (**d**) qRT-PCR was used to detect the expression of the following tumor-related endothelial cell markers: TEM5 and TEM7 in MMECs and HUVECs. (**e**) The ability of MMECs and HUVECs to secrete VEGFA was detected by ELISA. *p < 0.05; **p < 0.01; ***p < 0.001.

We collected the supernatant of RPMI-8226 cells, prepared conditioned medium (CM), and cultured HUVECs for 24 h before labeling the obtained cells as MMECs. The results of our CCK-8 proliferation assay (Fig. 3a), transwell invasion assay (Fig. 3b), and transwell migration assay (Fig. 3c) indicated that MMECs exhibited stronger proliferation, invasion, and migration abilities than HUVECs (*p* < 0.01). The results of a quantitative reverse transcription polymerase chain reaction (qRT-PCR) assay showed that the expression of the tumor-related endothelial cell markers TEM 5 and TEM 7 in MMECs was significantly higher than that in HUVECs (Fig. 3d) (*p* < 0.001). Enzyme-linked immunosorbent assay (ELISA) results showed that the vascular endothelial growth factor A (VEGFA) content of the MMEC supernatant was significantly higher than that of the HUVEC supernatant (Fig. 3e) (*p* < 0.05). The MMECs were therefore successfully induced and obtained.

### CAF promotion of MM angiogenesis through exosomes

**Figure 4:**
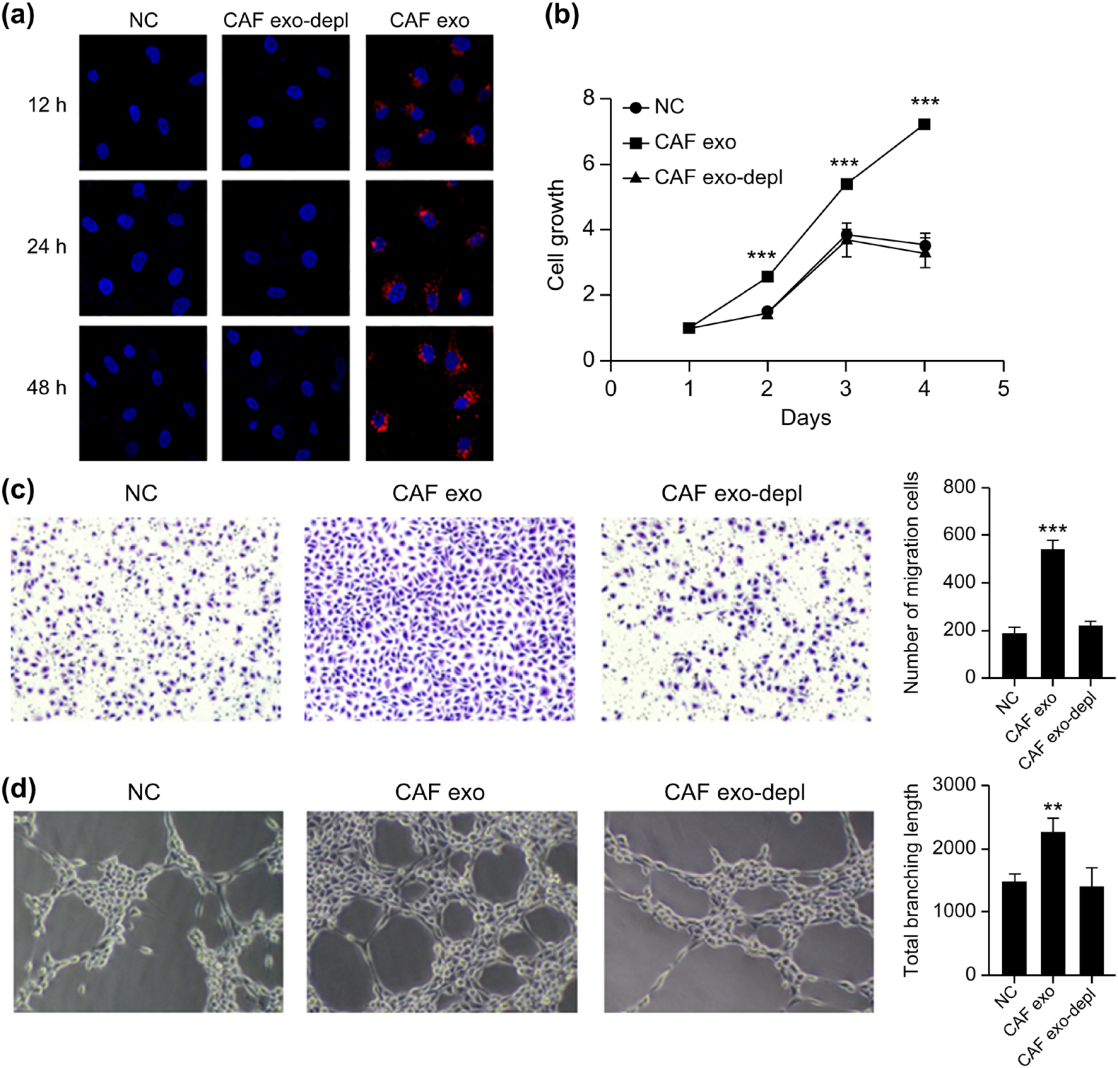
The effect of CAF-derived exosomes on MMECs. (**a**) The uptake of CAF exosomes by MMECs was observed by confocal microscopy (630 ×) (CAF exosomes marked in red and MMEC nuclei marked in blue); (**b**) CCK-8, (**c**) transwell migration assay, and (**d**) tubule formation assay were used to detect the effects of CAF exosomes and supernatant without exosomes on the proliferation, migration (100 ×), and tubule formation (100 ×) abilities of MMECs, respectively. **p < 0.01; ***p < 0.001.

To analyze whether CAF-derived exosomes could be transferred to MMECs, PKH26-labeled exosomes were co-cultured with MMECs and their nuclei were stained with 4′,6-diamidino-2-phenylindole (DAPI) after 12 h, 24 h, and 48 h, consecutively. Confocal microscopy showed that the PKH26-labelled exosomes gradually entered the MMEC cells and were eventually distributed around the nucleus. However, no red fluorescence was observed in the cytoplasm of the MMECs co-cultured with CAF supernatant without exosomes (Fig. 4a). These results indicated that CAF exosomes could be taken up by MMECs.

Next, we evaluated whether exosomes derived from CAFs affected the proliferation, migration, and tubular formation capacity of MMECs. The MMECs co-cultured with CAF exosomes and CAF supernatant without exosomes were labeled as the CAF exo group and the CAF exo-depl group, respectively. MMECs that had been cultured separately were labeled as the NC group. The results showed that the cells in the CAF exo group had a significant increase in proliferation (Fig. 4b) (*p* < 0.001), migration (Fig. 4c) (*p* < 0.001), and tubular formation capacity (Fig. 4d) (*p* < 0.01) compared to those in the NC group. However, the CAF exo-depl group did not differ significantly from the NC group. These results suggest that CAFs exosomes can promote angiogenesis in MMECs by promoting their proliferation, migration, and tubular formation abilities.

### CAF exosomes promote MM angiogenesis through miR-21

**Figure 5:**
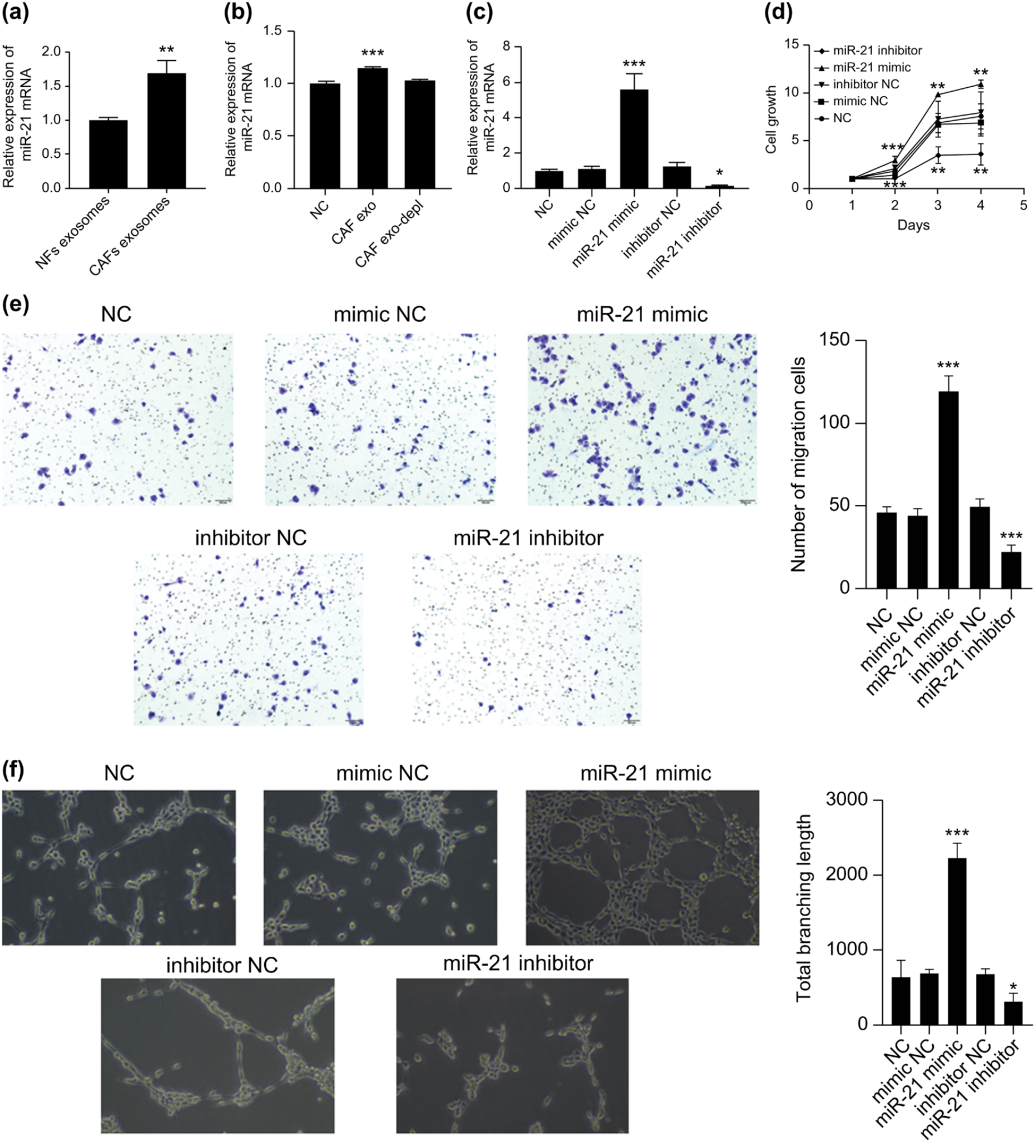
The effect of regulating miR-21 expression on E-MMECs. (**a**) The expression of miR-21 in NF and CAF exosomes was detected by qRT-PCR; (**b**) expression of miR-21 in MMECs cultured with CAF exosomes and supernatant without exosomes; and (**c**) expression of miR-21 in E-MMECs in each group after transfection. (**d**) CCK-8, (**e**) transwell migration test, and (**f**) tubule formation test were used to detect the proliferation, migration (100 ×), and tubule formation (100 ×) ability of E-MMECs in each group, respectively. *p < 0.05; **p < 0.01; ***p < 0.001.

Based on previous studies, we detected miR-21 expression in NFs and CAFs exosomes, and our qRT-PCR results showed that the expression of miR-21 in CAF exosomes was significantly higher than that in NF exosomes (Fig. 5a) (*p* < 0.01). Therefore, we further explored the effect of CAF exosomal miR-21 on MM angiogenesis.

Firstly, qRT-PCR was used to detect the effect of CAF exosomes and CAF supernatant (with exosomes removed) on the expression of miR-21 in MMECs after culturing for 48 h. As shown in Fig. 5b, compared to the NC group, expression of miR-21 in the cells of the CAF exo group was significantly increased, to approximately 1.15 times that of the NC group (*p* < 0.001). However, there was no significant difference between the CAF exo-depl and NC groups. These results suggest that CAFs exosomes can carry miR-21 into MMECs. In addition, we measured the miR-21 levels in HUVECs and MMECs using qRT-PCR, which showed no significant difference between the two groups (Fig. S2). Thus, we excluded the effect of RPMI-8226 supernatant on miR-21 levels in HUVECs. The MMECs of CAF exosomes cultured for 48 h were denoted as E-MMECs. The E-MMECs were transfected with either a mimic NC, a miR-21 mimic, an inhibitor NC, or a miR-21 inhibitor (Fig. 5c) (*p* < 0.05). These were labeled as mimic NC, miR-21 mimic, inhibitor NC, and miR-21 inhibitor groups. Un-transfected E-MMECs were labeled as the NC group. The results showed that 48 h after transfection, the proliferation (Fig. 5d) (*p* < 0.01), migration (Fig. 5e) (*p* < 0.001), and tubular formation (Fig. 5f) (*p* < 0.001) abilities of cells in the miR-21 mimic group were significantly higher than those in the NC group, whereas the opposite was true in the miR-21 inhibitor group (Fig. 5d,e) (*p* < 0.05). These results suggest that the regulation of miR-21 can affect the effect of CAF-derived exosomes on MMECs; that is, that CAFs promote angiogenesis in MM tumors through exosomal miR-21.

### The miR-21 transforms fibroblasts into CAFs

**Figure 6:**
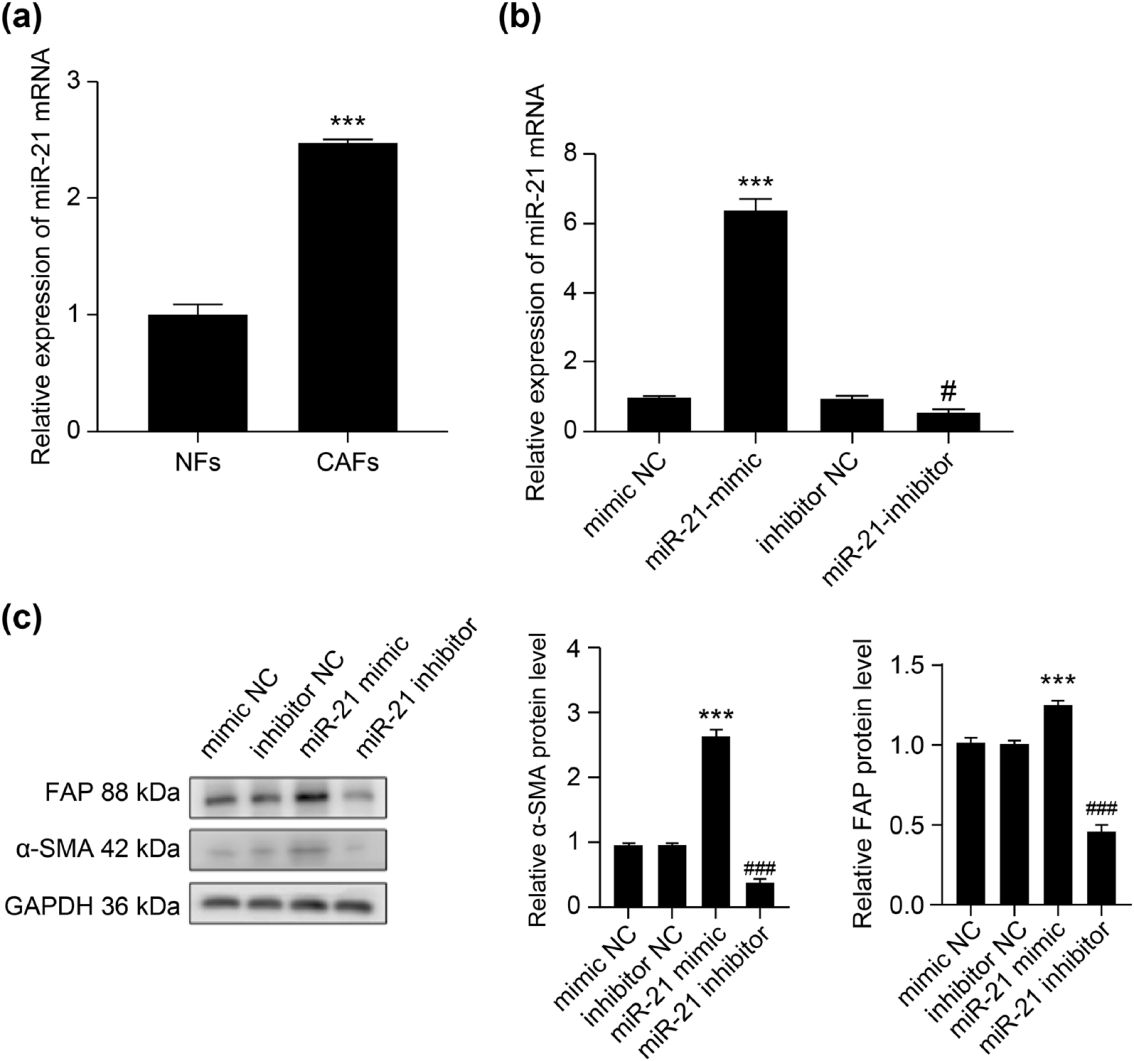
The effect of regulating miR-21 expression on the activation of NFs into CAFs. (**a**) The expression of miR-21 in NFs and CAFs was detected by qRT-PCR. (**b**) Relative expression of miR-21 in the NFs of each group. (**c**) The relative expression of α-SMA and FAP proteins in NFs was detected by Western blot. ***p < 0.001; #*p* < 0.05; ##*p* < 0.01; ###*p* < 0.0001 (*compared to the mimic NC group; # compared to the inhibitor NC group).

To investigate the effect of miR-21 on the conversion of NFs to CAFs, we examined the expression of miR-21 in both cell types. The qRT-PCR results showed that miR-21 expression was significantly higher in CAFs (Fig. 6a) (*p* < 0.001). Next, we transfected either a mimic NC, a miR-21 mimic, an inhibitor NC, or a miR-21 inhibitor into NFs (Fig. 6b) (*p* < 0.05). WB results showed that the miR-21 mimic significantly promoted the expression of α-SMA (Fig. 6c) (*p* < 0.001) and FAP (Fig. 6c) (*p* < 0.001) in NFs, compared to the NC mimic (*p* < 0.05). Compared to the NC inhibitor, the miR-21 inhibitor significantly inhibited the expression of α-SMA (Fig. 6c) (*p* < 0.01) and FAP (Fig. 6c) (*p* < 0.001) in NFs. These results suggest that miR-21 activates NFs to become CAFs.

## Discussion

Abnormal proliferation and infiltration of myeloma cells can cause extensive osteolytic bone destruction and a series of clinical symptoms such as functional decline and anemia, due to extramedullary infiltration of myeloma and renal involvement. Angiogenesis, in cases of MM, participates in this process and determines its progression, response to treatment, and the prognosis of the disease^6^. Previous studies have highlighted the ability of VEGF to promote tumor angiogenesis^35^. The humanized recombinant monoclonal antibody bevacizumab can inhibit tumor angiogenesis and delay tumor growth and metastasis by specifically binding to VEGF ^36^. Bevacizumab has become the first-line treatment for colorectal cancer, cervical cancer, glioblastoma, and other tumors, and has achieved satisfactory results^37–39^. However, angiogenesis in MM is a complex process, and the use of angiogenesis-targeting combination regimens with bevacizumab directly acting on VEGF is affected by multiple factors. Therefore, there is an urgent need to find new and potentially more effective ways to inhibit angiogenesis in MM. In this study, we found that CAFs were associated with MM angiogenesis, providing evidence that CAF-generated exosomal miR-21 mediates angiogenesis. Moreover, we discovered the role of miR-21 in the activation of NFs to form CAFs in MM.

Interregulation between TAMs, CAFs, tumor-associated endothelial cells, and other interstitial cells in the tumor microenvironment, as well as between interstitial cells and tumor cells, is involved in tumor progression^40,41^. As the most important tumor microenvironment components, CAFs can promote the development of malignant tumors. In the early stages of tumors, NFs can inhibit tumorigenesis. During tumor development, NFs are irreversibly transformed into CAFs, promoting tumor development in a variety of ways^42–45^. Several studies have revealed an interaction between CAFs and MM cells. Zi et al.^46^ found that bone marrow mononuclear cells (BMMCs) could be transformed into CAFs in tumor medium, or co-cultured with myeloma cell lines, and that CAFs could inhibit the apoptosis of MM cells and further promote drug resistance in MM cells, possibly through the activation of the β-catenin signaling pathway. Frassanito et al.^47^ found that MM tumor cells could induce CAF proliferation, which in turn induced MM tumor cell adhesion, proliferation, and inhibition of apoptosis. However, studies have shown that CAFs not only promote tumor proliferation and migration, but also participate in tumor angiogenesis by secreting pro-angiogenic factors, recruiting endothelial progenitor cells, regulating extracellular matrix remodeling, and by other mechanisms^48–50^. Currently, there are few studies on the relationship between CAFs and angiogenesis in MM. Therefore, we explored the pathway by which CAFs regulate angiogenesis in MM.

Studies have shown that blood vessels in tumors are morphologically and functionally very different from normal blood vessels. Morphologically, the vascular structure of tumors is disorganized, the basement membrane is abnormal, endothelial cells do not form a monolayer, and pericyte cytoplasm extends into the tumor tissue. Functionally, blood vessels in tumors may collapse, obstruct blood flow, lose barrier function, and increase permeability^51^. The blood vessels in tumor tissues are composed of tumor-associated endothelial cells^52^. In this study, to better simulate the MM tumor microenvironment, HUVECs were induced to become MMECs, after which the angiogenesis of MM was investigated through the proliferation, migration, and tubular formation abilities of MMECs, rather than that of HUVECs. Compared to that of HUVECs, the MMEC phenotype is more inclined toward tumor-associated endothelial cells, and its proliferation, migration, and tubular formation abilities are significantly higher than those of HUVECs. In addition, the angiogenesis-related gene VEGFA was significantly expressed in the supernatant of MMECs. This was further confirmed by Vacca et al.’s comparison study of primary MMECs and HUVECs^53^. As observed, the use of MMECs in in vitro studies can better simulate the tumor microenvironment, thereby improving the authenticity and reliability of the experiment.

In this study, we isolated and characterized exosomes from CAFs and found that these exosomes could be transferred into MMECs in a time-dependent manner. Notably, we found that CAF exosomes not only promoted the proliferation and migration capacity of MMECs, but also significantly enhanced their tubular formation capacity. Exosomes contain biological macromolecules such as DNA, RNA, proteins, and lipids^54^. Studies have shown that exosomal miRNAs can interact with endothelial cells through multiple mechanisms to promote tumor angiogenesis. Wu et al.^55^ showed that hypoxic papillary thyroid cancer cells can regulate angiogenesis through exosome-mediated miR-21-5p/TGFBI and miR-21-5p/COL4A1 signaling pathways. Umezu et al.^56^ believed that the HR-MM cell-derived exosome miR-135b could promote the formation of endodermal tubes through the HIF-FIH signaling pathway under hypoxic conditions.

Based on previous studies, we further analyzed the expression of the angiogenesis-related miRNA, miR-21, in CAFs exosomes. We co-incubated CAFs exosomes with MMECs for at least 48 h and detected the expression of miR-21 in MMECs using qRT-PCR. Our results showed that the expression of miR-21 was significantly increased in MMECs after co-incubation with CAF exosomes. One of the first non-coding RNAs discovered in the human genome was miR-21, which is closely related to the activation, proliferation, migration, apoptosis, and angiogenesis of vascular endothelial cells, and can increase the number and density of blood vessels in vivo. An et al.^57^ showed that miR-21 overexpression in adipose-derived stem cells promoted the vascularization of HUVECs via exosomes. He et al.^58^ found that colorectal cancer cells (CRC) were capable of transferring miR-21-5p into HUVECs via exosomes, to subsequently induce CRC angiogenesis and vascular permeability by inhibiting KRIT1 and activating the β-catenin signaling pathway. In this study, to better demonstrate the role of CAF exosome-derived miR-21 in MM angiogenesis, we used a NC mimic, a miR-21 mimic, a NC inhibitor, and a miR-21 inhibitor to transfect MMECs co-incubated with CAF exosomes for 48 h (labelled “E-MMECs”). We observed that the miR-21 mimic significantly enhanced the proliferation, migration, and tubular formation abilities of E-MMECs. However, the miR-21 inhibitor had the opposite effect. Therefore, we concluded that CAF exosome-derived miR-21 can enhance the angiogenic capacity of MM cells.

Numerous studies have reported that miR-21 is highly expressed in CAFs. In their study on CAFs and lung adenocarcinoma progression, Kunita et al.^14^ found that miR-21 was highly expressed in CAFs, and that it correlated with poor prognoses in lung adenocarcinoma cases. In addition, Chen et al.^59^ found that the expression of miR-21 was elevated in CAFs, compared to NFs. Furthermore, they reported that miR-21 can affect the development of pancreatic cancer cells by regulating the metabolisms of CAFs. In this study, for the first time, miR-21 levels in both the NFs and CAFs of patients with dystrophic anemia and MM were examined. We found that miR-21 levels were higher in CAFs than in NFs. By regulating the expression of miR-21 in NFs, we found that high miR-21 expression significantly upregulated the expression of α-SMA and FAP in NFs. This suggests that miR-21 activates NFs into CAFs. Previous studies have shown that miRNAs can promote CAF activation. Zhou et al.^60^ showed that melanoma cells can reduce SOCS1 expression through secreted exosomal miR-155, which in turn triggers the activation of NFs into pro-angiogenic CAFs by activating the JAK2/STAT3 signaling pathway. Cheng et al.^61^ found that MM partially promotes the proliferation, CAF transformation, and interleukin-6 secretion of mesenchymal stem cells (MSCs) by regulating miR-21 and miR-146a. Zhou et al.^11^ found that hepatocellular carcinoma-derived exosomal miRNA-21 can convert hepatocyte stellate cells into CAFs and promote tumor progression. Our findings further confirmed the tumor-promoting role of miR-21 in MM. This may be due to its ability to transform NFs into CAFs, thereby regulating angiogenesis.

The high expression of miR-21 in CAFs is associated with multiple factors including tumor cells and their microenvironment. Kunita et al.^14^ found that CM from lung cancer cells can increase the expression of miR-21 in lung fibroblasts through the TGF-β pathway and induce the formation of CAFs. Cheng et al.^61^ showed that MM cell exosomes contain high levels of miR-21. Co-culture with MSCs can increase the proliferation ability of MSCs, upregulate the level of miR-21 in MSCs, and induce the transformation of MSCs into CAFs. Therefore, in MM, it is necessary to further explore the specific mechanism of miR-21 overexpression in CAFs. The target genes of miR-21 include PDCD4, SMAD7, PTEN, HIF-1α, KRIT1, and other tumor suppressor genes. Zhang et al.^15^ found that miR-21 promotes the activation of CAFs in pancreatic cancer by regulating the expression of PDCD4. Li et al.^62^ reported that, along with TGF-β, miR-21 promoted CAF activation by binding to the 3′ UTR of Smad7. Liu et al.^33^ found that miR-21 induced HIF-1α and VEGF expression through the PTEN/AKT/ERK signaling pathway, thereby promoting angiogenesis in prostate cancer. He et al.^58^ claimed that exocrine miR-21 derived from colorectal cancer cells can promote tumor angiogenesis by targeting KRIT1 in HUVECs. However, the molecular mechanism by which miR-21 in MM activates NFs to form CAFs, and the pathway by which miR-21 and CAFs promotes the angiogenesis of MM have not been clarified and requires further exploration.

This study had certain limitations. The tumor microenvironment of MM is more hypoxic than that of other solid tumors, and the degree of hypoxia is not only related to the progression and prognosis of MM but is also closely related to angiogenesis^63–65^. Numerous studies have shown that hypoxia promotes the release of exosomes from cells. Panigrahi et al.^66^ found that PCa cells secreted more exosomes under chronic hypoxic conditions, in order to remove metabolic waste. Xi et al.^67^ found that oxidized ATM induced autophagosome accumulation and exosome release in hypoxic mammary CAFs by BNIP3 phosphorylation. The expression of miR-21, a hypoxia-related miRNA, was significantly increased under hypoxic conditions. Through miRNA sequencing, Li et al.^68^ showed that miR-21 was significantly upregulated in exosomes derived from anoxic oral squamous cell carcinoma. Liu et al.^69^ showed that hypoxia can induce the expression of miR-21 in a MM cell line (U266) and that miR-21 can promote the survival of MM cells by targeting PTEN. Chang et al.^70^ found that hypoxia can induce the expression of miR-21 in HUVECs. Therefore, analyzing the effect of miR-21 in CAF-derived exosomes on MM angiogenesis both in vitro and in vivo, under hypoxic conditions, may be able to better demonstrate the important role of exosomal miR-21 in the occurrence and development of MM.

Taken together, our findings highlight the important roles of miR-21, CAFs, and CAF exosomes in MM angiogenesis. The miR-21 induces CAF activation and activated CAFs in turn support MM angiogenesis by releasing miR-21-enriched exosomes to promote MMEC proliferation, migration, and tube formation. As these effects are mediated by miR-21, exosomes have long half-lives, high stabilities, easy separation, and long-term persistence. Therefore, targeting miR-21 in CAF exosomes can be envisioned as a novel and potentially effective approach to inhibit MM angiogenesis.

## Materials and methods

### Patients and cell culture

Fresh bone marrow fluid was collected from dystrophic anemia (n = 6) and MM (n = 13) patients in the Hematology Department of the First Affiliated Hospital of Zhengzhou University from December 2019 to August 2021 for primary cell isolation. All patients with MM were newly diagnosed and had not received chemoradiotherapy or immunotherapy before the study was conducted. This study was reviewed and approved by the Life Science Ethics Review Committee of Zhengzhou University. All participating patients provided signed informed consent. All methods were performed in accordance with the relevant guidelines and regulations.

The human MM cell line RPMI-8226 and HUVECs were cultured in Iscove's modified Dulbecco's media and RPMI-1640 (Gibco, New York, USA) supplemented with 10% fetal bovine serum (Gibco, New York, USA) and 1% penicillin–streptomycin solution.

### Isolation and culture of fibroblasts

BMMCs were isolated from the patients’ bone marrow fluids using Ficoll density gradient centrifugation. BMMCs were cultured in Dulbecco's modified eagle medium (DMEM; Gibco, New York, USA) supplemented with 20% fetal bovine serum (Gibco, New York, USA) and 1% penicillin–streptomycin solution. Fibroblasts were then purified from BMMCs using three different adherents.

### Immune cell fluorescence

The purity of the NFs and CAFs was verified using immunofluorescence detection of α-SMA and FAP. Cells were seeded on the cover glass of a circular microscope. After 24 h, the cells were fixed with 4% paraformaldehyde universal tissue fixative for 30 min, permeabilized with 0.5% Triton X-100 (Shangbao, Shanghai, China) for 20 min, and blocked with a goat serum working solution for 30 min. The cells were then incubated with anti-α-SMA (1:200; Abcam) or anti-FAP (1:200; Bioss) antibodies overnight at 4 °C. After incubation with the goat anti-rabbit IgG (1:1000, Abcam), the cells were observed and photographed under an upright fluorescence microscope.

### WB

The expression of α-SMA, FAP, glyceraldehyde 3-phosphate dehydrogenase (GAPDH), CD63, and CD81 proteins were detected by WB. Total protein was extracted from cells or exosomes using RIPA reagent (Invitrogen, USA), according to the manufacturer’s instructions. The protein samples from each group were added to the loading wells, and electrophoresis was completed when the bromophenol blue indicator migrated to the bottom of the separation rubber. A piece of polyvinylidene fluoride film slightly larger than the separation glue was cut, and the film was spread flat on the glue for film turning. After the transfer was completed, the cells were blocked for 2 h. The PVDF membrane was cut into appropriate sizes and placed in 15 ml centrifuge tubes containing anti α-SMA (1:1000, Abcam), anti FAP (1:1000, Abcam), anti GAPDH (1:10,000, Abcam), anti CD63 (1:1000, Abcam), or anti CD81 (1:1000, Abcam) antibodies followed by overnight incubation at 4℃. The next day, after incubating the secondary antibody, appropriate amount of ECL working solution was added, and the multi-functional gel imaging system was used for development.

### Induction and culture of MMECs

RPMI-8226 cells were inoculated into 75 L flasks at 5 × 10^5^/mL. When the cells reached the exponential growth period, the supernatant was collected, centrifuged at 300 rpm for 10 min, and mixed with DMEM at a ratio of 4:6 to prepare a CM. HUVECs were cultured in the conditioned medium and induced to become MMECs after 24 h. The functions of the induced MMECs were also verified.

### Cell proliferation test

The cells were seeded in 96-well plates and tested according to CCK-8 reagent instructions. The absorbance of the cells at the 490 nm wavelength was detected using a multifunctional microplate reader for 4 or 5 days.

### Cell migration assay

- *Transwell chamber migration assay* the medium containing 20% fetal bovine serum was added to a 24-well plate at 600 µL/well. The chamber was then placed in a 24-well plate. The cells were seeded into the chamber at a density of 1 × 10^5^ cells/well. After 18–24 h, the bottom of the chamber was fixed with 4% paraformaldehyde for 30 min, stained with 0.1% crystal violet for 20 min, washed with phosphate-buffered saline (PBS), dried, and photographed under a microscope to evaluate the migration abilities of the cells in each group.
- *Scratch test* the cells were seeded in 6-well plates at a density of 5 × 10^5^ cells/well. When the cells were full, a pipette gun tip was used to draw a horizontal line perpendicular to the back of the 6-well plate. The floating cells were washed with PBS, and basal medium was added for further culturing. Cellular migration in each group was observed and photographed at 12, 24, 36, and 48 h, consecutively.

### Transwell invasion assay

The medium containing 20% fetal bovine serum was added to the 24-well plate (600 µL/well). The transwell chamber was transferred to the 24-well plate, and 50 μL of diluted matrix gel (BD Biosciences, Franklin Lakes, NJ, USA) was added to the bottom of the chamber. The cells were resuspended in basal medium and seeded in the chamber at a density of 5 × 10^5^ cells/well. They were then treated identically to those of the transwell chamber migration assay.

### Tube formation test

The tube formation ability of the cells was tested using a tube formation test. Diluted matrigel (250 μL; BD Biosciences, Franklin Lakes, NJ, USA) was added to a 24-well plate and polymerized for 40 min. Next, the cells were resuspended in the basal medium and seeded in 24-well plates at a density of 1 × 10^5^ cells/well. After 4 h, the angiogenesis of the cells in each group was observed and photographed under an inverted microscope.

### ELISA

The cell supernatant was collected and centrifuged at 2000 rpm for 5 min, then measured using human VEGFA ELISA kits (RayBiotech, Atlanta, GA, USA) according to the manufacturer's instructions.

### Exosomal isolation from NF and CAF media

At 70–80% cell confluence, the basal medium was replaced for cell culturing and the NF and CAF supernatants were collected after another 48 h. Cells were removed by centrifugation at 2000 rpm for 5 min, and cellular debris was removed using a 0.22-μm filter. Exosomes were then isolated from the supernatant using the Exoquick-TC kit (SBI, CA, USA). The obtained exosomes were resuspended in PBS and stored at − 80 °C after aliquotting. A bicinchoninic acid kit was used to determine the concentration of exosomes. To analyze the effect of CAF exosomes on MMECs, 2 µg of exosomes was added to 1 × 10^5^ MMECs for at least 48 h.

### Transmission electron microscope analysis

After 1 min, 20 μL of exosomes were added to a copper net dropwise, the excess liquid at the edge was absorbed by a filter paper, and a solution of dioxouranium acetate was added to the net for negative staining and fixing for 1–10 min. The excess liquid at the edge was absorbed by a filter paper, dried naturally, observed, and photographed under an HT-7700 electron microscope (Hitachi, Tokyo, Japan).

### NTA

Exosomal size and concentration were measured using ZetaView PMX 110 (Particle Metrix, Meerbusch, Germany) and ZetaView 8.04.02. Exosome samples were then diluted in 1X PBS buffer (Biological Industries, Israel), recorded, and analyzed at 11 locations. The ZetaView system was calibrated using 110 nm polystyrene particles. Temperatures were maintained at approximately 23–30 °C.

### PKH26 staining of exosomes

Firstly, 100 μL of PBS-exosome suspension was added to 100 µL of Diluent C and thoroughly mixed to prepare liquid A; 0.5 μL of PKH26 stock solution was then added to 100 µL of Diluent C and thoroughly mixed to prepare liquid B. Thereafter, liquid A and liquid B were mixed (using an Eppendorf tube and avoiding light), shaken at room temperature for 5 min, added to 200 µL of 5% bovine serum albumin solution to stop staining, and placed in a 4 °C refrigerator overnight. Finally, after centrifugation at 1500 rpm at 4 °C for 30 min, the red precipitate at the bottom of the tube was the exosomes labeled with PKH26.

### RNA isolation and qRT-PCR

Total RNA was extracted using Trizol reagent (Invitrogen, USA) and mRNA and miRNA were quantified using a SYBR probe (Vazyme, Nanjing, China). After the reaction, the threshold cycle (CT) values of each group were analyzed using the 2-delta-delta CT (2-∆∆CT) relative quantification method to obtain the relative number of target genes. U6 and GAPDH were used as the internal parameters for miRNA and mRNA, respectively. Primers for miR-21, TEM5, TEM7, U6, and GAPDH were designed as summarized in Table 1.

**Table 1:** Primer sequences.

Primer	Sequence	
miR-21	Forward	5′-GCGCGTAGCTTATCAGACTGA-3′
	Reverse	5′-AGTGCAGGGTCCGAGGTATT-3′
U6	Forward	5′-CTCGCTTCGGCAGCACA-3′
	Reverse	5′-AACGCTTCACGAATTTGCGT-3′
TEM5	Forward	5′-CCACAGCCACAGCAACACCTC-3′
	Reverse	5′-CCACTGGTTCCTGCGAAGATGAC-3′
TEM7	Forward	5′-TGCGAGGACTTCCAGGATGAGG-3′
	Reverse	5′-AGGGAGGAGGAGGTAGTGGTGAG-3′
GAPDH	Forward	5′-CTTAGCACCCCTGGCCAAG-3′
	Reverse	5′-GATGTTCTGGAGAGCCCCG-3′

### Cell transfection

Cells were seeded and transfected with riboFE^CT^TMCP buffer and riboFE^CT^TMCP reagent (RiboBio, Guangzhou, China) according to the manufacturer’s instructions. For upregulation and downregulation of miRNA, 50 nM of mimic NC and miR-21 mimic, and 100 nM of inhibitor NC and miR-21 inhibitor were used. Cells were then collected for qRT-PCR analysis 48 h after transfection, or after 72 h for WB analysis. The sequences of our mimic NC, miR-21 mimic, inhibitor NC, and miR-21 inhibitor are summarized in Table 2.

**Table 2:** Sequences of mimic NC, miR-21 mimic, inhibitor NC, and miR-21 inhibitor.

Sequence number	Sequence	
Mimic NC	Sense:	5′-UUUGUACUACACAAAAGUACUG-3′
	Antisense:	5′-CAGUACUUUUGUGUAGUACAAA-3′
miR-21 mimic	Sense:	5′-UAGCUUAUCAGACUGAUGUUGA-3′
	Antisense:	5′-UCAACAUCAGUCUGAUAAGCUA-3′
Inhibitor NC	Sense:	5′-CAGUACUUUUGUGUAGUACAAA-3′
miR-21 inhibitor	Sense:	5′-UCAACAUCAGUCUGAUAAGCUA-3′

### Data analysis

All experiments were repeated at least three times. SPSS 21.0 statistical software was used for statistical analysis. The mean and standard deviation were used to describe the measurement data, independent sample t-test was used to analyze the comparison between the two groups, and one-way analysis of variance was used to analyze the comparison between multiple groups. Differences were considered statistically significant at *p* < 0.05.

## Supplementary Information

Supplementary Information.

## Data Availability

The datasets generated or analyzed during this study are included in this publication.
