# Supplementary material for: Cancer-associated fibroblast-derived exosome microRNA-21 promotes angiogenesis in multiple myeloma
Source: Sci Rep. 2023 Jun 14;13:9671. doi: 10.1038/s41598-023-36092-6 (PMC10267152; doi:10.1038/s41598-023-36092-6)

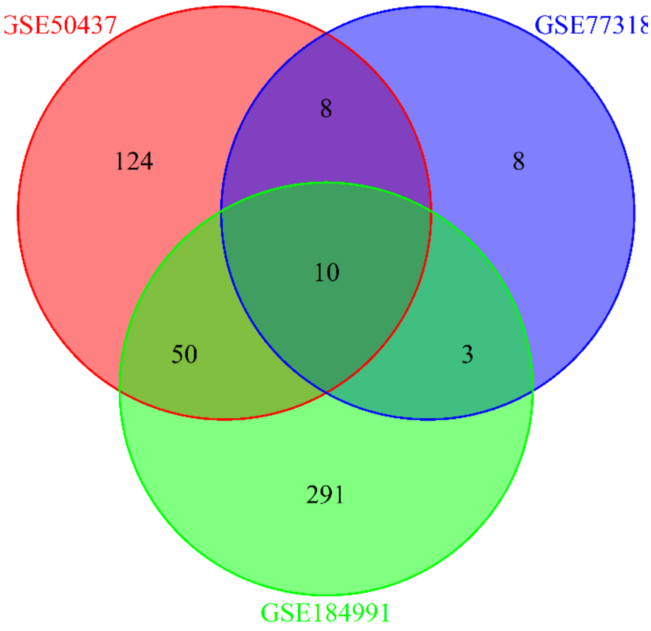

Fig. S1. MiR-21 is one of the most closely related miRNAs to angiogenesis in CAFs and CAF exosomes.

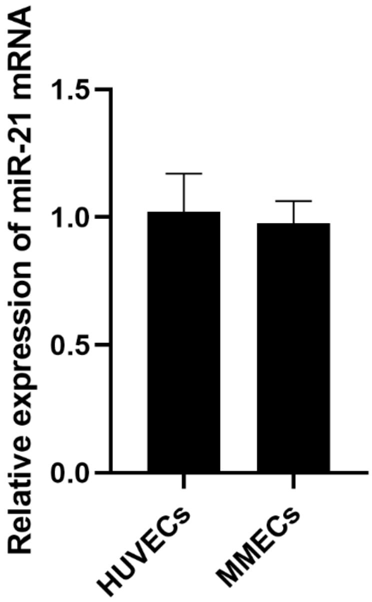

Fig. S2. The expression of miR-21 in HUVECs and MMECs was detected by qRT-PCR.

List of miRNAs in the common parts of the Venn diagram.

GSE50437	GSE77318	GSE184991	..count..	values
TRUE	TRUE	TRUE	10	hsa-miR-21, hsa-miR-195, hsa-miR-31, hsa-miR-23b, hsa-miR-376a, hsa-miR-30c, hsa-miR-181a, hsa-let-7g, hsa-miR-379, hsa-miR-143
FALSE	TRUE	TRUE	3	hsa-miR-329, hsa-let-7b, hsa-miR-378d
TRUE	FALSE	TRUE	50	hsa-miR-27b, hsa-miR-29b, hsa-miR-29c, hsa-miR-20a, hsa-miR-432, hsa-miR-193a, hsa-miR-194, hsa-miR-100, hsa-miR-126, hsa-miR-99a, hsa-miR-30a, hsa-miR-222, hsa-let-7c, hsa-miR-197, hsa-miR-211, hsa-miR-101, hsa-miR-30d, hsa-miR-26a, hsa-miR-525, hsa-miR-149, hsa-let-7f, hsa-miR-214, hsa-miR-196b, hsa-miR-99b, hsa-miR-423, hsa-miR-296, hsa-let-7a, hsa-miR-485, hsa-miR-140, hsa-miR-199a, hsa-miR-335, hsa-miR-202, hsa-miR-328, hsa-miR-136, hsa-miR-504, hsa-miR-338, hsa-miR-493, hsa-miR-30e, hsa-miR-187, hsa-miR-134, hsa-miR-199b, hsa-miR-95, hsa-miR-518c, hsa-miR-154, hsa-miR-34a, hsa-miR-223, hsa-miR-373, hsa-miR-382, hsa-miR-412, hsa-miR-218
FALSE	FALSE	TRUE	291	hsa-let-7e, hsa-miR-1, hsa-miR-1180, hsa-miR-1182, hsa-miR-1197, hsa-miR-1204, hsa-miR-1224, hsa-miR-1225, hsa-miR-1226, hsa-miR-1228, hsa-miR-1229, hsa-miR-1236, hsa-miR-1238, hsa-miR-1247, hsa-miR-1248, hsa-miR-1250, hsa-miR-1253, hsa-miR-125a, hsa-miR-125b, hsa-miR-1268a, hsa-miR-1268b, hsa-miR-1269a, hsa-miR-127, hsa-miR-1271, hsa-miR-1281, hsa-miR-1282, hsa-miR-1286, hsa-miR-1296, hsa-miR-1304, hsa-miR-1323, hsa-miR-133a, hsa-miR-135a, hsa-miR-135b, hsa-miR-139, hsa-miR-145, hsa-miR-1468, hsa-miR-147b, hsa-miR-1539, hsa-miR-1587, hsa-miR-1825, hsa-miR-18b, hsa-miR-190a, hsa-miR-1913, hsa-miR-1915, hsa-miR-204, hsa-miR-2054, hsa-miR-2117, hsa-miR-2276, hsa-miR-2355, hsa-miR-2681, hsa-miR-26b, hsa-miR-299, hsa-miR-302d, hsa-miR-3122, hsa-miR-3123, hsa-miR-3124, hsa-miR-3129, hsa-miR-3131, hsa-miR-3135b, hsa-miR-3150b, hsa-miR-3155a, hsa-miR-3155b, hsa-miR-3160, hsa-miR-3190, hsa-miR-3195, hsa-miR-320a, hsa-miR-320b, hsa-miR-320d, hsa-miR-323a, hsa-miR-33b, hsa-miR-3607, hsa-miR-3612, hsa-miR-3613, hsa-miR-3622a, hsa-miR-3622b, hsa-miR-3667, hsa-miR-3670, hsa-miR-3677, hsa-miR-3681, hsa-miR-369, hsa-miR-3713, hsa-miR-375, hsa-miR-376c, hsa-miR-377, hsa-miR-378a, hsa-miR-378c, hsa-miR-378e, hsa-miR-378f, hsa-miR-378g, hsa-miR-378h, hsa-miR-378i, hsa-miR-381, hsa-miR-3911, hsa-miR-3915, hsa-miR-3936, hsa-miR-3937, hsa-miR-

				3944, hsa-miR-3972, hsa-miR-3974, hsa-miR-409, hsa-miR-411, hsa-miR-421, hsa-miR-422a, hsa-miR-4251, hsa-miR-4253, hsa-miR-4258, hsa-miR-4259, hsa-miR-4265, hsa-miR-4290, hsa-miR-4297, hsa-miR-4303, hsa-miR-4320, hsa-miR-4322, hsa-miR-4324, hsa-miR-4328, hsa-miR-4417, hsa-miR-4436a, hsa-miR-4442, hsa-miR-4444, hsa-miR-4448, hsa-miR-4472, hsa-miR-4479, hsa-miR-4481, hsa-miR-4486, hsa-miR-4487, hsa-miR-4493, hsa-miR-4507, hsa-miR-4510, hsa-miR-4523, hsa-miR-4524a, hsa-miR-4532, hsa-miR-4538, hsa-miR-4646, hsa-miR-4647, hsa-miR-4655, hsa-miR-4656, hsa-miR-4658, hsa-miR-4664, hsa-miR-4670, hsa-miR-4681, hsa-miR-4687, hsa-miR-4700, hsa-miR-4713, hsa-miR-4718, hsa-miR-4725, hsa-miR-4734, hsa-miR-4735, hsa-miR-4738, hsa-miR-4740, hsa-miR-4746, hsa-miR-4748, hsa-miR-4749, hsa-miR-4750, hsa-miR-4751, hsa-miR-4761, hsa-miR-4768, hsa-miR-4769, hsa-miR-4778, hsa-miR-4781, hsa-miR-4783, hsa-miR-4794, hsa-miR-4796, hsa-miR-4797, hsa-miR-483, hsa-miR-487b, hsa-miR-497, hsa-miR-499a, hsa-miR-499b, hsa-miR-5000, hsa-miR-5006, hsa-miR-500a, hsa-miR-5011, hsa-miR-508, hsa-miR-510, hsa-miR-5186, hsa-miR-5189, hsa-miR-518e, hsa-miR-520d, hsa-miR-548an, hsa-miR-548ao, hsa-miR-548q, hsa-miR-548u, hsa-miR-551b, hsa-miR-5572, hsa-miR-5587, hsa-miR-5685, hsa-miR-5701, hsa-miR-574, hsa-miR-578, hsa-miR-582, hsa-miR-589, hsa-miR-595, hsa-miR-596, hsa-miR-6068, hsa-miR-6072, hsa-miR-6080, hsa-miR-6084, hsa-miR-611, hsa-miR-6128, hsa-miR-613, hsa-miR-6165, hsa-miR-622, hsa-miR-623, hsa-miR-628, hsa-miR-634, hsa-miR-646, hsa-miR-647, hsa-miR-6501, hsa-miR-6508, hsa-miR-6510, hsa-miR-6511a, hsa-miR-6511b, hsa-miR-654, hsa-miR-659, hsa-miR-664a, hsa-miR-664b, hsa-miR-668, hsa-miR-6715b, hsa-miR-6729, hsa-miR-6732, hsa-miR-6739, hsa-miR-6745, hsa-miR-6747, hsa-miR-6753, hsa-miR-6755, hsa-miR-6764, hsa-miR-6766, hsa-miR-6768, hsa-miR-6775, hsa-miR-6781, hsa-miR-6786, hsa-miR-6788, hsa-miR-6792, hsa-miR-6796, hsa-miR-6797, hsa-miR-6798, hsa-miR-6802, hsa-miR-6804, hsa-miR-6805, hsa-miR-6808, hsa-miR-6809, hsa-miR-6816, hsa-miR-6817, hsa-miR-6819, hsa-miR-6823, hsa-miR-6827, hsa-miR-6828, hsa-miR-6830, hsa-miR-6836, hsa-miR-6844, hsa-miR-6846, hsa-miR-6851, hsa-miR-6857, hsa-miR-6859, hsa-miR-6861, hsa-miR-6862, hsa-miR-6863, hsa-miR-6864, hsa-miR-6865, hsa-miR-6871, hsa-miR-6872, hsa-miR-6874, hsa-miR-6875, hsa-miR-6876, hsa-miR-6882, hsa-miR-6885, hsa-miR-6888, hsa-miR-6891, hsa-miR-6894, hsa-miR-7108, hsa-miR-7109, hsa-miR-7114, hsa-miR-7156, hsa-miR-

				7158, hsa-miR-7161, hsa-miR-744, hsa-miR-758, hsa-miR-7641, hsa-miR-765, hsa-miR-766, hsa-miR-7846, hsa-miR-8066, hsa-miR-8073, hsa-miR-8078, hsa-miR-8079, hsa-miR-8085, hsa-miR-874, hsa-miR-875, hsa-miR-891a, hsa-miR-937, hsa-miR-98
TRUE	TRUE	FALSE	8	hsa-miR-15b, hsa-miR-221, hsa-miR-30b, hsa-miR-153, hsa-miR-452, hsa-miR-330, hsa-miR-494,
FALSE	TRUE	FALSE	8	hsa-miR-3960, hsa-miR-103a, hsa-miR-93, hsa-miR-210, hsa-miR-940, hsa-miR-590, hsa-miR-625, hsa-miR-484
TRUE	FALSE	FALSE	124	hsa-miR-29a, hsa-miR-27a, hsa-miR-106a, mmu-miR-106a, hsa-miR-190, hsa-miR-192, hsa-miR-103, hsa-miR-10a, hsa-miR-17, hsa-miR-10b, hsa-miR-186, hsa-miR-24, hsa-miR-33, hsa-miR-22, hsa-miR-492, hsa-miR-19a, hsa-miR-34b, hsa-miR-16, hsa-miR-196a, rno-miR-347, hsa-miR-183, hsa-miR-331, hsa-miR-18a, hsa-miR-19b, hsa-miR-23a, hsa-miR-105, hsa-miR-188, hsa-miR-107, hsa-miR-301, mmu-miR-293, hsa-miR-138, hsa-let-7i, hsa-miR-345, mmu-miR-17, hsa-miR-380, mmu-miR-202, hsa-miR-200b, hsa-miR-185, hsa-miR-142, hsa-miR-368, hsa-miR-28, rno-miR-346, mmu-miR-192, 0--, hsa-miR-212, mmu-miR-383, hsa-miR-141, hsa-miR-15a, hsa-miR-449, hsa-miR-526b, hsa-miR-181b, hsa-miR-152, rno-miR-421, hsa-miR-106b, mmu-miR-215, hsa-miR-193b, rno-miR-151, hsa-miR-182, rno-miR-333, hsa-miR-184, mmu-miR-199b, hsa-miR-340, mmu-miR-297, hsa-miR-7, rno-miR-297, hsa-miR-346, hsa-miR-500, hsa-miR-410, hsa-miR-499, hsa-miR-324, hsa-miR-206, hsa-miR-216, hsa-miR-144, hsa-miR-215, mmu-miR-350, mmu-miR-201, hsa-miR-523, mmu-miR-298, hsa-miR-425, hsa-miR-181c, hsa-miR-518d, mmu-miR-376a, hsa-miR-189, hsa-miR-501, mmu-miR-330, hsa-miR-323, hsa-miR-326, hsa-miR-519e, hsa-miR-520b, hsa-miR-25, hsa-miR-450, hsa-miR-422b, hsa-miR-370, hsa-miR-520a, hsa-miR-489, hsa-miR-491, hsa-miR-122a, hsa-miR-132, hsa-miR-515, hsa-miR-512, mmu-miR-337, hsa-miR-198, mmu-miR-384, hsa-miR-429, hsa-miR-513, mmu-let-7d, hsa-miR-325, rno-miR-336, hsa-miR-155, mmu-miR-291, hsa-miR-527, hsa-miR-496, hsa-miR-320, hsa-miR-524, hsa-miR-448, mmu-miR-290, hsa-miR-147, mmu-miR-217, hsa-miR-517, rno-miR-327, hsa-miR-509, hsa-miR-502, hsa-miR-208, hsa-miR-383

Partial miRNA list in dataset GSE50437, GSE184991 and GSE77318.

GSE50437	P.Value	GSE184991	P.Value	GSE77318	P.Value
hsa-miR-21	0.000000	hsa-let-7c-5p	0.000046	hsa-miR-330	0.000110
hsa-miR-29a	0.000000	hsa-miR-99a-3p	0.000087	hsa-miR-3960	0.001074

hsa-miR-27a	0.000000	hsa-miR-125b-2-3p	0.000095	hsa-miR-329-1	0.001118
hsa-miR-27b	0.000000	hsa-miR-4324	0.000149	hsa-miR-103a-2	0.001223
hsa-miR-29b	0.000000	hsa-miR-99a-5p	0.000164	hsa-miR-143	0.002046
hsa-miR-29c	0.000000	hsa-miR-125b-5p	0.000167	hsa-miR-181a-1	0.003558
hsa-miR-106a	0.000000	hsa-miR-139-5p	0.000176	hsa-miR-93	0.005133
hsa-miR-15b	0.000000	hsa-miR-6510-3p	0.000208	hsa-miR-210	0.005438
hsa-miR-20a	0.000000	hsa-miR-199b-5p	0.000224	hsa-miR-153-1	0.005733
hsa-miR-195	0.000000	hsa-miR-6804-3p	0.000266	hsa-miR-940	0.006006
hsa-miR-221	0.000000	hsa-miR-30a-3p	0.000446	hsa-miR-31	0.012408
hsa-miR-31	0.000000	hsa-miR-125a-5p	0.000469	hsa-miR-30c-2	0.015495
mmu-miR-106a	0.000000	hsa-miR-2117	0.000525	hsa-miR-23b	0.016815
hsa-miR-432	0.000000	hsa-miR-204-5p	0.000572	hsa-miR-590	0.021899
hsa-miR-193a	0.000000	hsa-miR-145-5p	0.000636	hsa-miR-21	0.023699
hsa-miR-194	0.000000	hsa-miR-499a-5p	0.000668	hsa-let-7b	0.023816
hsa-miR-100	0.000000	hsa-miR-149-5p	0.000689	hsa-miR-494	0.024254
hsa-miR-190	0.000000	hsa-miR-8073	0.000732	hsa-miR-15b	0.024582
hsa-miR-192	0.000000	hsa-miR-126-5p	0.000823	hsa-miR-221	0.024650
hsa-miR-103	0.000000	hsa-miR-154-5p	0.000872	hsa-miR-625	0.025160
hsa-miR-126	0.000000	hsa-miR-223-3p	0.001253	hsa-miR-452	0.030947
hsa-miR-10a	0.000000	hsa-miR-6511a-3p	0.001322	hsa-miR-30b	0.035365
hsa-miR-99a	0.000000	hsa-miR-190a-3p	0.001367	hsa-let-7g	0.039144
hsa-miR-30a-5p	0.000000	hsa-miR-504-5p	0.001410	hsa-miR-195	0.040673
hsa-miR-17-5p	0.000000	hsa-miR-30a-5p	0.001419	hsa-miR-379	0.042029
hsa-miR-10b	0.000000	hsa-miR-483-3p	0.001499	hsa-miR-181a-2	0.044046
hsa-miR-30b	0.000000	hsa-miR-6501-3p	0.001539	hsa-miR-484	0.045182
hsa-miR-23b	0.000000	hsa-miR-4442	0.001556	hsa-miR-378d-2	0.048485
hsa-miR-376a	0.000000	hsa-miR-379-5p	0.001627	hsa-miR-376a-1	0.049696
hsa-miR-222	0.000000	hsa-miR-7641	0.001652	hsa-miR-326	0.051554
hsa-miR-17-3p	0.000000	hsa-miR-187-3p	0.001654	hsa-let-7c	0.053856
hsa-miR-186	0.000000	hsa-miR-766-5p	0.001677	hsa-miR-107	0.056522
hsa-miR-24	0.000000	hsa-miR-6755-3p	0.001770	hsa-miR-1306	0.057248
hsa-let-7c	0.000001	hsa-miR-218-5p	0.001930	hsa-miR-323a	0.057248
hsa-miR-33	0.000001	hsa-miR-382-3p	0.002105	hsa-miR-409	0.058097
hsa-miR-197	0.000001	hsa-miR-654-3p	0.002123	hsa-miR-182	0.059599
hsa-miR-22	0.000001	hsa-miR-328-3p	0.002159	hsa-miR-3591	0.062301
hsa-miR-492	0.000001	hsa-miR-1-5p	0.002181	hsa-miR-136	0.062305
hsa-miR-19a	0.000001	hsa-miR-483-5p	0.002250	hsa-miR-212	0.062305
hsa-miR-34b	0.000001	hsa-miR-214-3p	0.002262	hsa-miR-497	0.062305
hsa-miR-211	0.000001	hsa-miR-485-5p	0.002329	hsa-miR-224	0.062359
hsa-miR-16	0.000002	hsa-miR-296-5p	0.002367	hsa-miR-1260a	0.068074
hsa-miR-101	0.000002	hsa-miR-4681	0.002440	hsa-miR-574	0.070033
hsa-miR-196a	0.000002	hsa-miR-6080	0.002509	hsa-miR-487b	0.070131
hsa-miR-30c	0.000002	hsa-miR-378h	0.002616	hsa-miR-145	0.073923

hsa-miR-30d	0.000002	hsa-miR-376c-3p	0.002628	hsa-let-7a-1	0.075854
rno-miR-347	0.000002	hsa-miR-101-3p	0.002640	hsa-miR-32	0.077682
hsa-miR-126-AS	0.000003	hsa-miR-378e	0.002785	hsa-miR-376c	0.078869
hsa-miR-26a	0.000005	hsa-miR-574-3p	0.002877	hsa-miR-451a	0.079007
hsa-miR-183	0.000005	hsa-miR-1182	0.002878	hsa-miR-337	0.080161
hsa-miR-331	0.000006	hsa-miR-1247-5p	0.003024	hsa-miR-1273a	0.086574
hsa-miR-18a	0.000006	hsa-miR-6068	0.003066	hsa-miR-29c	0.086574
hsa-miR-525-AS	0.000006	hsa-miR-6128	0.003101	hsa-miR-744	0.093027
hsa-miR-181a	0.000007	hsa-miR-4507	0.003266	hsa-let-7a-2	0.093758
hsa-miR-19b	0.000008	hsa-let-7e-3p	0.003309	hsa-miR-1285-1	0.099886
hsa-miR-149	0.000009	hsa-miR-6816-5p	0.003350	hsa-miR-19a	0.103247
hsa-let-7f	0.000011	hsa-miR-3123	0.003357	hsa-miR-7-1	0.103398
hsa-miR-23a	0.000011	hsa-miR-378f	0.003359	hsa-miR-342	0.103786
hsa-miR-105	0.000012	hsa-miR-3622b-3p	0.003529	hsa-miR-30a	0.106088
hsa-miR-188	0.000013	hsa-miR-4646-5p	0.003708	hsa-miR-495	0.107986
hsa-miR-214	0.000016	hsa-miR-329-3p	0.003807	hsa-miR-381	0.109849
hsa-miR-196b	0.000016	hsa-miR-127-3p	0.003881	hsa-miR-15a	0.110238
hsa-miR-107	0.000019	hsa-miR-1296-5p	0.004042	hsa-miR-130b	0.110899
hsa-miR-301	0.000022	hsa-miR-6844	0.004057	hsa-miR-502	0.113136
hsa-miR-99b	0.000022	hsa-miR-1248	0.004079	hsa-miR-19b-1	0.115353
hsa-miR-423	0.000025	hsa-miR-199b-3p	0.004096	hsa-miR-576	0.116402
hsa-miR-296	0.000027	hsa-miR-195-5p	0.004278	hsa-miR-205	0.116406
hsa-let-7a	0.000030	hsa-miR-190a-5p	0.004278	hsa-miR-421	0.116584
mmu-miR-293	0.000037	hsa-miR-7158-5p	0.004328	hsa-miR-664	0.118830
hsa-miR-138	0.000037	hsa-miR-373-5p	0.004464	hsa-miR-152	0.119369
hsa-let-7i	0.000038	hsa-miR-518c-5p	0.004489	hsa-miR-222	0.125546
hsa-miR-345	0.000044	hsa-miR-381-3p	0.004498	hsa-miR-485	0.127775
mmu-miR-17-3p	0.000045	hsa-let-7e-5p	0.004643	hsa-miR-1228	0.127870
hsa-miR-380-5p	0.000049	hsa-miR-1587	0.004683	hsa-miR-601	0.127870
mmu-miR-202	0.000050	hsa-miR-378a-3p	0.004701	hsa-miR-17	0.131732
hsa-let-7g	0.000056	hsa-miR-4328	0.004983	hsa-miR-197	0.134801
hsa-miR-200b	0.000058	hsa-miR-139-3p	0.005000	hsa-miR-126	0.137123
hsa-miR-185	0.000059	hsa-miR-299-5p	0.005020	hsa-miR-140	0.140145
hsa-miR-142-3p	0.000060	hsa-miR-126-3p	0.005173	hsa-miR-106b	0.146362
hsa-miR-368	0.000067	hsa-miR-6885-5p	0.005277	hsa-miR-148a	0.154924
hsa-miR-379	0.000082	hsa-miR-100-5p	0.005302	hsa-miR-132	0.157579
hsa-miR-28	0.000092	hsa-miR-98-5p	0.005323	hsa-miR-18a	0.158886
hsa-miR-485-5p	0.000104	hsa-miR-3129-5p	0.005450	hsa-miR-199a-1	0.160080
rno-miR-346	0.000108	hsa-miR-1228-3p	0.005558	hsa-miR-361	0.178894
mmu-miR-192	0.000117	hsa-miR-199a-3p	0.005564	hsa-miR-148b	0.184957
0	0.000125	hsa-miR-140-5p	0.005599	hsa-miR-183	0.184957
hsa-miR-140	0.000158	hsa-miR-497-5p	0.005696	hsa-miR-202	0.187207
hsa-miR-143	0.000165	hsa-miR-5189-3p	0.006121	hsa-miR-5095	0.187207

hsa-miR-212	0.000172	hsa-miR-3936	0.006167	hsa-miR-301a	0.187509
hsa-miR-199a-AS	0.000173	hsa-miR-204-3p	0.006186	hsa-miR-101-1	0.190588
mmu-miR-383	0.000189	hsa-miR-143-3p	0.006191	hsa-miR-26a-1	0.192482
hsa-miR-153	0.000198	hsa-miR-378i	0.006209	hsa-miR-92b	0.195200
hsa-miR-141	0.000206	hsa-miR-378a-5p	0.006211	hsa-miR-127	0.195945
hsa-miR-15a	0.000216	hsa-miR-6808-3p	0.006225	hsa-miR-4419a	0.196719
hsa-miR-335	0.000217	hsa-miR-369-3p	0.006358	hsa-miR-1180	0.199381
hsa-miR-452	0.000235	hsa-miR-29c-5p	0.006390	hsa-miR-1273d	0.199381
hsa-miR-449	0.000246	hsa-miR-3607-3p	0.006615	hsa-miR-4459	0.199381
hsa-miR-330	0.000294	hsa-miR-6788-3p	0.006648	hsa-miR-1296	0.199656
hsa-miR-526b	0.000304	hsa-miR-578	0.006880	hsa-miR-20a	0.202939
hsa-miR-202-AS	0.000325	hsa-miR-4417	0.006900	hsa-miR-24-1	0.203221
hsa-miR-328	0.000342	hsa-miR-595	0.006905	hsa-miR-99a	0.203494
hsa-miR-181b	0.000346	hsa-miR-6817-3p	0.007017	hsa-miR-532	0.213292
hsa-miR-136	0.000353	hsa-miR-2355-3p	0.007067	hsa-miR-184	0.219867
hsa-miR-152	0.000359	hsa-miR-4524a-3p	0.007133	hsa-miR-1254-1	0.224197
hsa-miR-452-AS	0.000389	hsa-miR-487b-3p	0.007359	hsa-miR-1268a	0.224197
hsa-miR-504	0.000420	hsa-miR-375	0.007469	hsa-miR-199b	0.224197
hsa-miR-338	0.000482	hsa-let-7g-5p	0.007731	hsa-miR-374a	0.224197
rno-miR-421	0.000578	hsa-miR-3713	0.008062	hsa-miR-193b	0.224963
hsa-miR-106b	0.000593	hsa-miR-664a-5p	0.008287	hsa-miR-1280	0.225008
mmu-miR-215	0.000624	hsa-miR-136-5p	0.008304	hsa-miR-1273e	0.228709
hsa-miR-193b	0.000665	hsa-miR-412-5p	0.008455	hsa-miR-1273g	0.228709
rno-miR-151-AS	0.000916	hsa-miR-4322	0.008463	hsa-miR-345	0.229372
hsa-miR-182-AS	0.001045	hsa-miR-193a-3p	0.008474	hsa-miR-186	0.229921
rno-miR-333	0.001154	hsa-miR-4436a	0.008718	hsa-miR-24-2	0.232821
hsa-miR-184	0.001175	hsa-miR-1-3p	0.008734	hsa-miR-500a	0.233293
mmu-miR-199b	0.001175	hsa-miR-758-5p	0.008869	hsa-miR-138-2	0.234857
hsa-miR-340	0.001233	hsa-miR-4444	0.008877	hsa-miR-1260b	0.236087
mmu-miR-297	0.001372	hsa-miR-582-5p	0.009061	hsa-miR-1301	0.239593
hsa-miR-7	0.001473	hsa-miR-622	0.009076	hsa-miR-324	0.240871
rno-miR-297	0.001526	hsa-miR-378c	0.009421	hsa-miR-16-1	0.244278
hsa-miR-346	0.001658	hsa-miR-376a-3p	0.009725	hsa-miR-4466	0.245006
hsa-miR-500	0.001770	hsa-miR-4751	0.009891	hsa-miR-27b	0.249614
hsa-miR-410	0.001904	hsa-miR-3150b-5p	0.009924	hsa-miR-3665	0.252611
hsa-miR-499	0.002111	hsa-let-7f-5p	0.010050	hsa-miR-4429	0.252611
hsa-miR-324-3p	0.002372	hsa-miR-1913	0.010176	hsa-miR-598	0.252611
hsa-miR-206	0.002397	hsa-let-7b-3p	0.010472	hsa-miR-551b	0.255001
hsa-miR-216	0.002587	hsa-miR-3972	0.010624	hsa-let-7d	0.257578
hsa-miR-144	0.002686	hsa-miR-1269a	0.010982	hsa-miR-196a-2	0.259603
hsa-miR-215	0.002778	hsa-miR-411-5p	0.011102	hsa-let-7f-2	0.265614
hsa-miR-493	0.002927	hsa-miR-1225-5p	0.011116	hsa-miR-1185-1	0.265614
hsa-miR-30e-5p	0.002990	hsa-miR-3974	0.011143	hsa-miR-1270-1	0.265614

mmu-miR-350	0.003131	hsa-miR-765	0.011172	hsa-miR-1278	0.265614
hsa-miR-187	0.003223	hsa-miR-6882-3p	0.011196	hsa-miR-4516	0.265614
hsa-miR-494	0.003627	hsa-miR-1236-5p	0.011267	hsa-miR-487a	0.265614
mmu-miR-201	0.003691	hsa-miR-5701	0.011651	hsa-miR-549	0.265614
hsa-miR-134	0.003992	hsa-miR-4320	0.011698	hsa-miR-95	0.265614
hsa-miR-523	0.004033	hsa-miR-6729-5p	0.011705	hsa-miR-134	0.265803
mmu-miR-298	0.004467	hsa-miR-26a-5p	0.011771	hsa-miR-181c	0.265803
hsa-miR-199b	0.004967	hsa-miR-4538	0.011841	hsa-miR-493	0.265803
hsa-miR-425	0.004989	hsa-miR-2054	0.011913	hsa-miR-543	0.265803
hsa-miR-181c	0.005186	hsa-miR-202-3p	0.011936	hsa-miR-656	0.265803
hsa-miR-518d	0.005461	hsa-miR-6819-5p	0.012099	hsa-miR-3117	0.265999
mmu-miR-376a	0.005484	hsa-miR-548q	0.012147	hsa-miR-33b	0.265999
hsa-miR-199a	0.005747	hsa-miR-3613-5p	0.012299	hsa-miR-3605	0.265999
hsa-miR-189	0.005818	hsa-miR-508-5p	0.012316	hsa-miR-3613	0.265999
hsa-miR-30a-3p	0.005900	hsa-miR-548u	0.012367	hsa-miR-382	0.265999
hsa-miR-501	0.006380	hsa-miR-647	0.012660	hsa-miR-567	0.265999
hsa-miR-30e-3p	0.006540	hsa-miR-29b-2-5p	0.012893	hsa-miR-654	0.265999
mmu-miR-330	0.006549	hsa-miR-6732-3p	0.012931	hsa-miR-663a	0.265999
hsa-miR-323	0.007038	hsa-miR-4740-5p	0.012956	hsa-miR-96	0.265999
hsa-miR-326	0.007558	hsa-miR-1229-5p	0.013095	hsa-miR-105-1	0.266558
hsa-miR-519e-AS	0.008082	hsa-miR-4510	0.013123	hsa-miR-141	0.266558
hsa-miR-520b	0.008268	hsa-miR-1281	0.013298	hsa-miR-154	0.266558
hsa-miR-25	0.009808	hsa-miR-26b-5p	0.013521	hsa-miR-188	0.266558
hsa-miR-450	0.009918	hsa-miR-6809-5p	0.013635	hsa-miR-3115	0.266558
hsa-miR-95	0.010146	hsa-miR-195-3p	0.013651	hsa-miR-34b	0.266558
hsa-miR-422b	0.010586	hsa-miR-4487	0.013787	hsa-miR-369	0.266558
hsa-miR-370	0.010845	hsa-miR-510-5p	0.013847	hsa-miR-370	0.266558
hsa-miR-520a-AS	0.011533	hsa-miR-891a-5p	0.013930	hsa-miR-376b	0.266558
hsa-miR-518c-AS	0.011549	hsa-miR-1180-5p	0.013943	hsa-miR-411	0.266558
hsa-miR-489	0.011743	hsa-miR-6753-5p	0.013968	hsa-miR-433	0.266558
hsa-miR-491	0.011933	hsa-miR-628-5p	0.013985	hsa-miR-4449	0.266558
hsa-miR-154	0.012627	hsa-miR-3677-5p	0.014052	hsa-miR-4536-1	0.266558
hsa-miR-122a	0.012810	hsa-miR-194-3p	0.014092	hsa-miR-4636	0.266558
hsa-miR-142-5p	0.012901	hsa-miR-4796-3p	0.014192	hsa-miR-4649	0.266558
hsa-miR-132	0.012944	hsa-miR-26a-2-3p	0.014223	hsa-miR-519c	0.266558
hsa-miR-34a	0.013653	hsa-miR-4258	0.014249	hsa-miR-128-1	0.273156
hsa-miR-515-3p	0.014036	hsa-miR-4781-5p	0.014359	hsa-miR-652	0.281213
hsa-miR-512-3p	0.014467	hsa-miR-125b-1-3p	0.014572	hsa-miR-29b-1	0.281315
mmu-miR-337	0.014718	hsa-miR-6830-5p	0.014587	hsa-miR-3676	0.288647
hsa-miR-198	0.015080	hsa-miR-421	0.014665	hsa-miR-191	0.302052
mmu-miR-384	0.015961	hsa-miR-21-5p	0.014682	hsa-miR-137	0.302728
hsa-miR-429	0.016376	hsa-miR-5186	0.014857	hsa-miR-374b	0.312899
hsa-miR-513	0.016564	hsa-miR-551b-3p	0.014863	hsa-let-7f-1	0.329698

hsa-miR-223	0.017005	hsa-miR-135a-5p	0.015184	hsa-miR-133a-1	0.330241

Raw, unprocessed data for all WB images in the article

Fig 1(c)-GAPDH

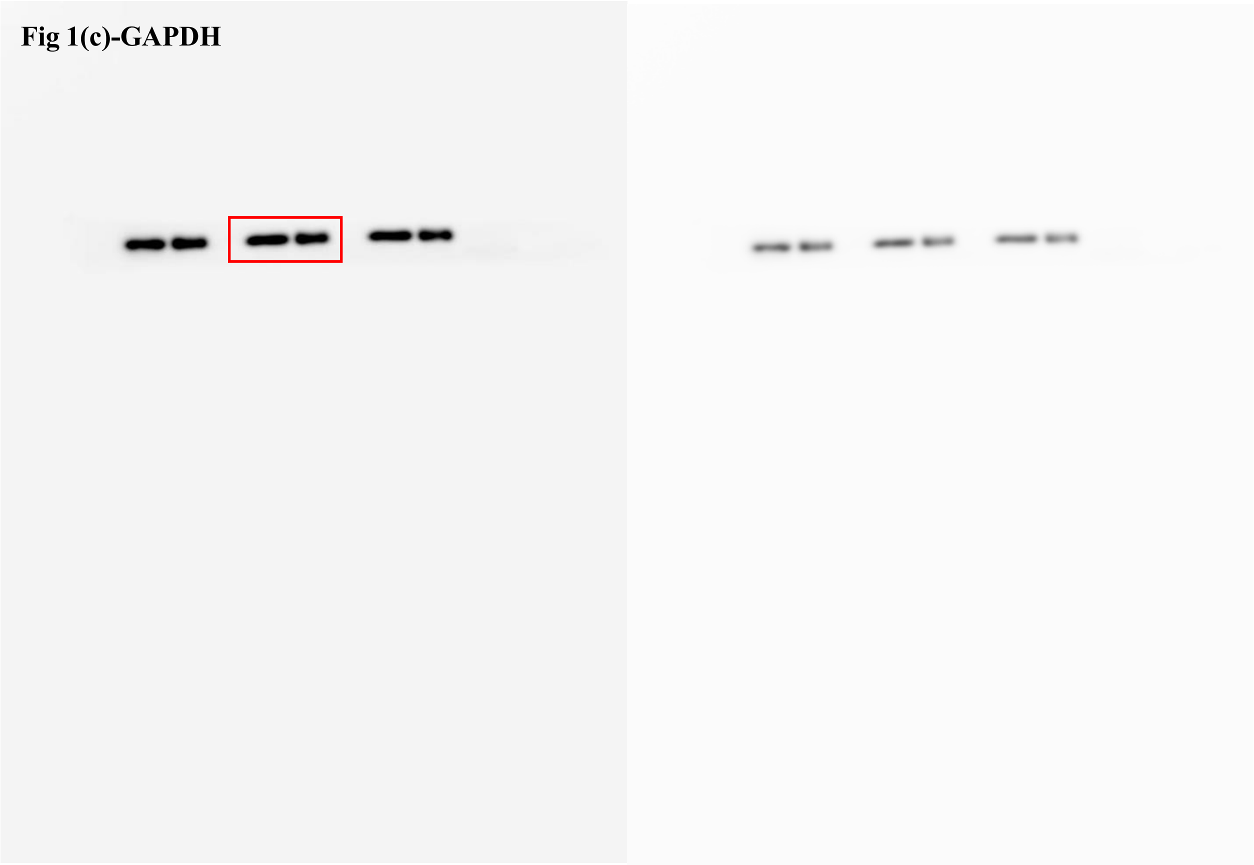

Fig 1(c)-α-SMA

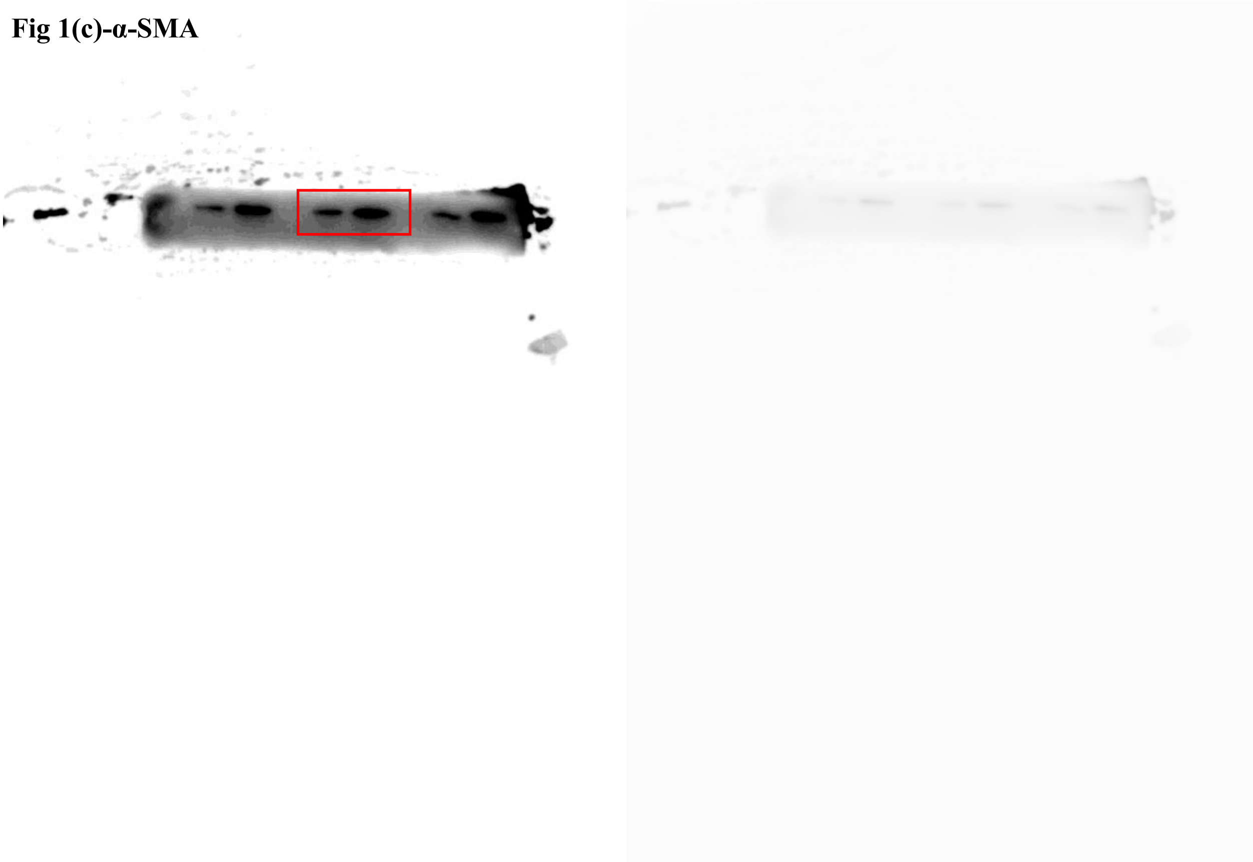

Fig 1(c)-FAP

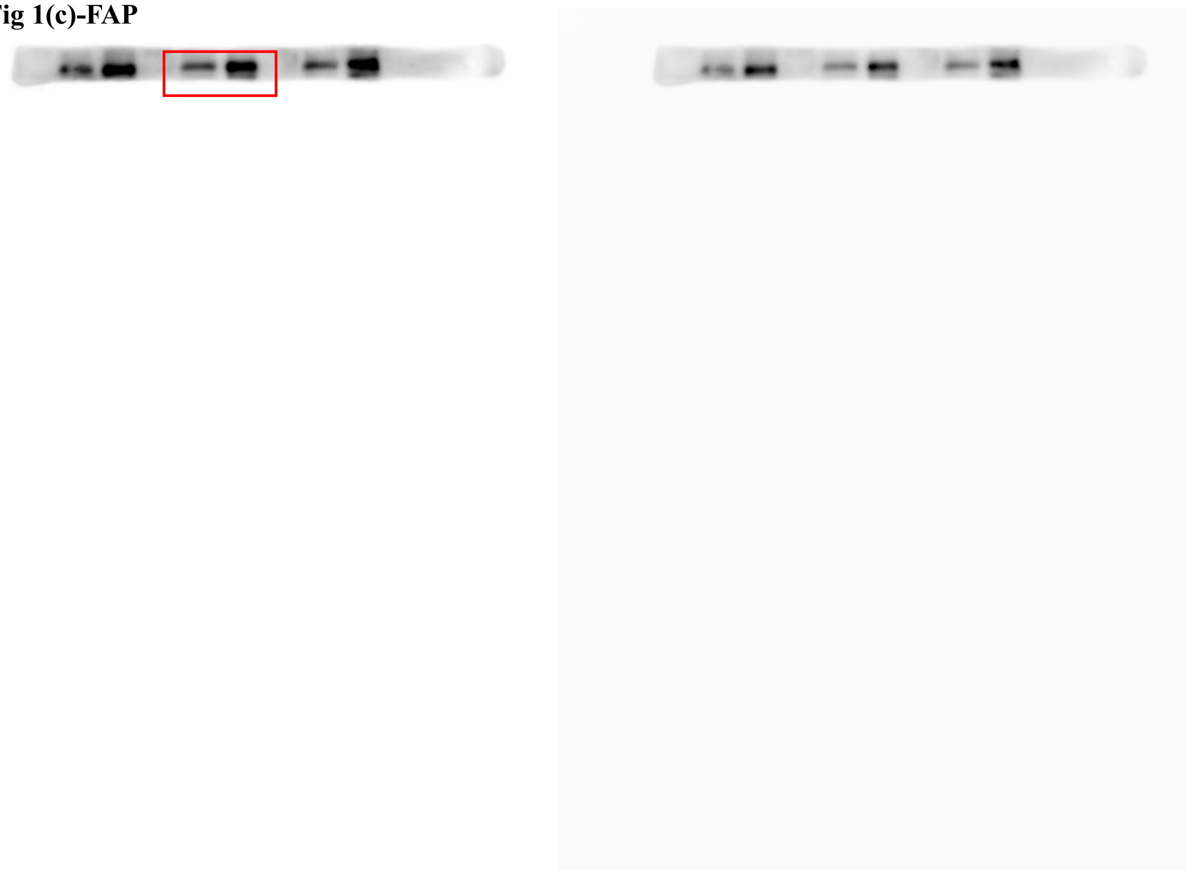

Fig 2(c)-CD63

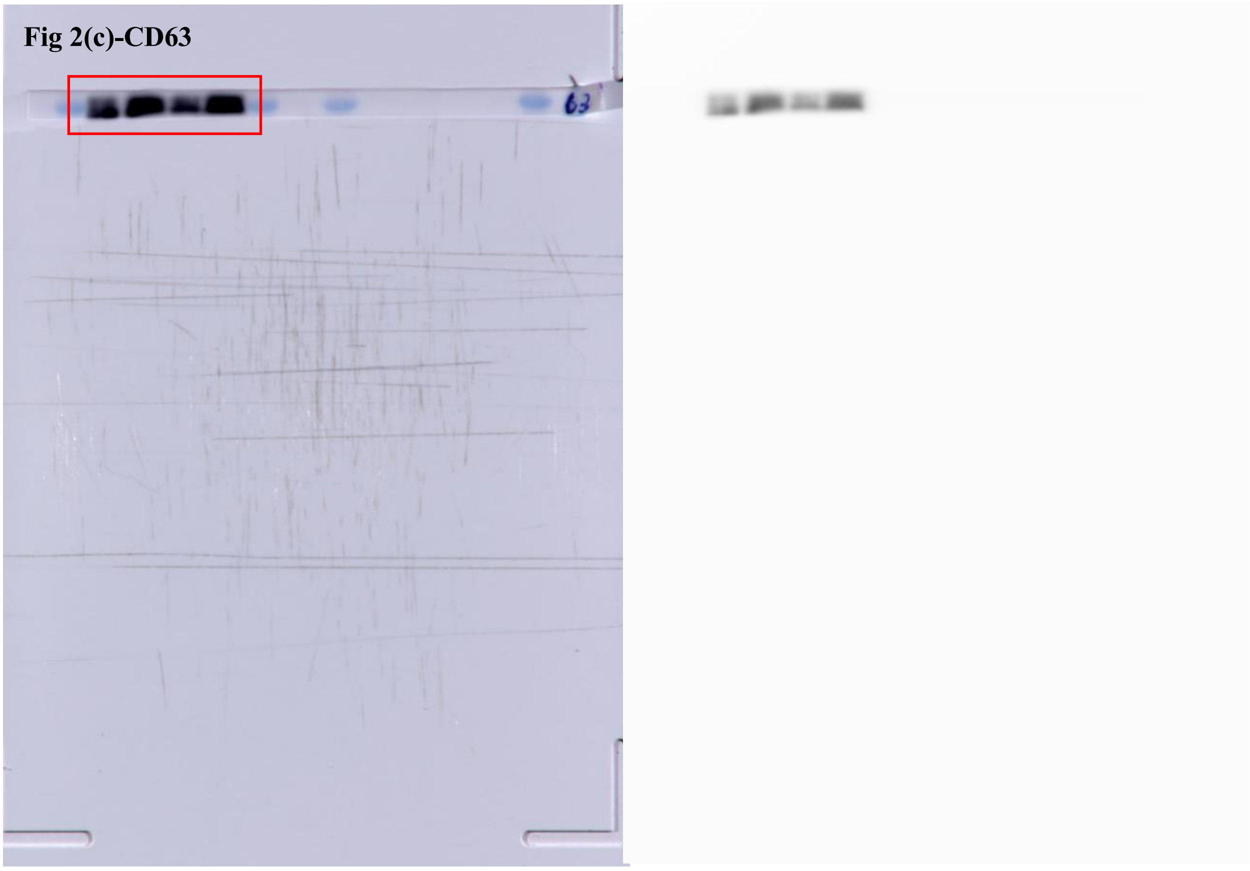

Fig 2(c)-CD81

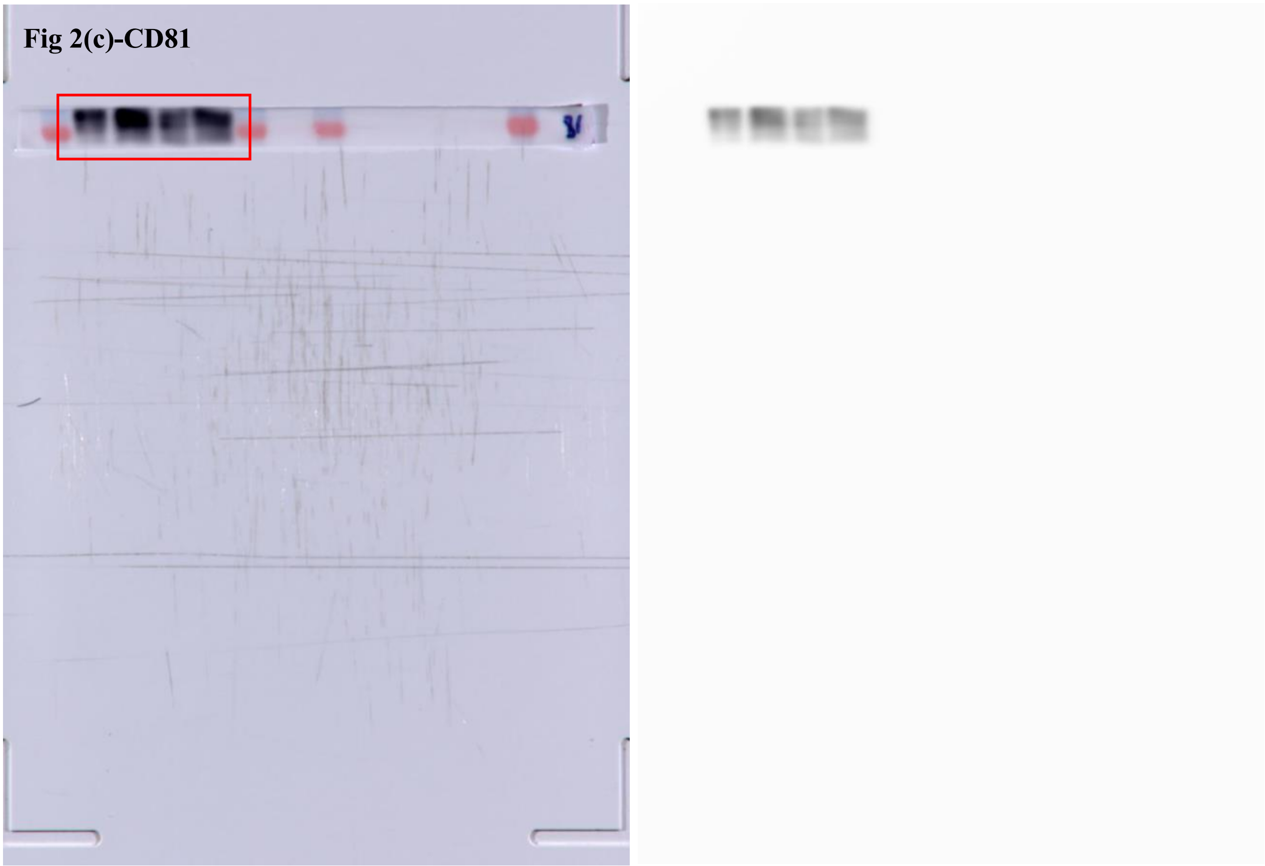

Fig 6(c)-GAPDH

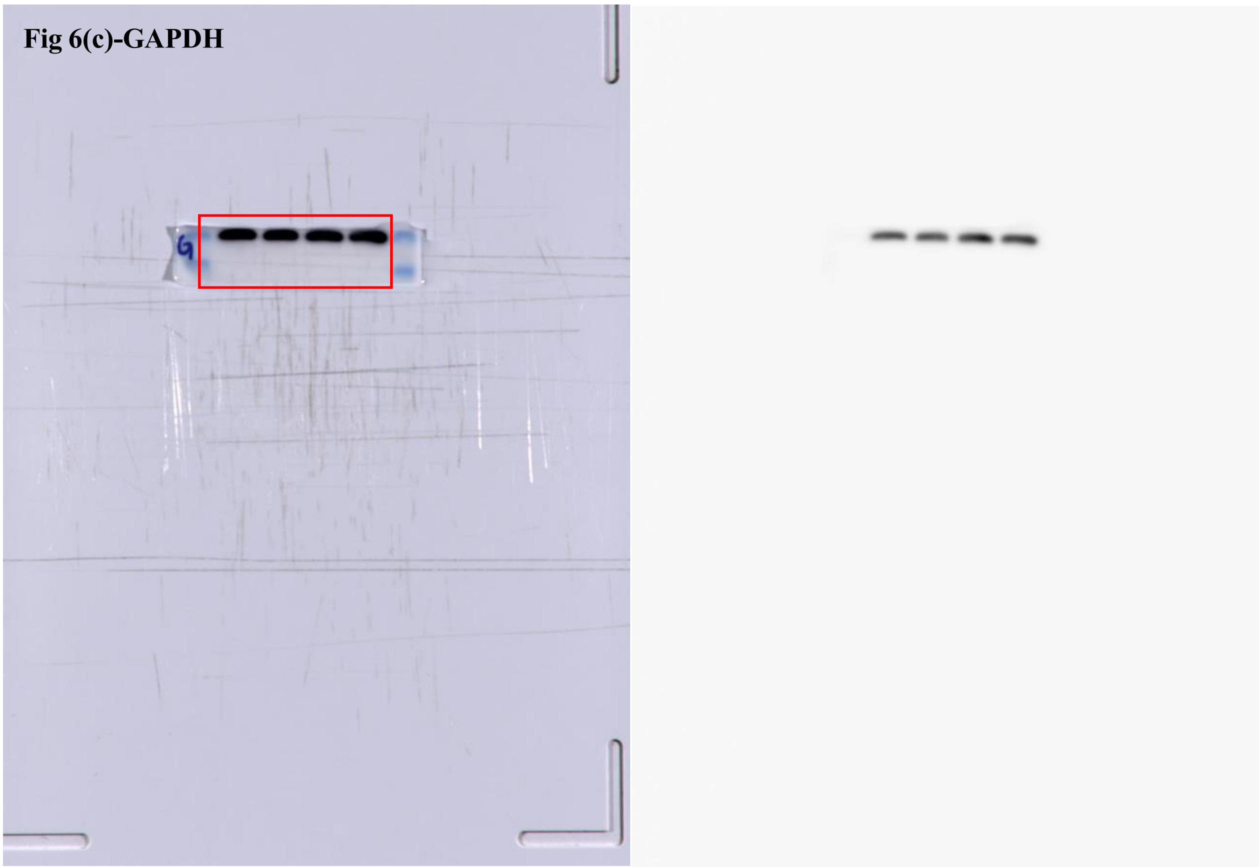

Fig 6(c)-α-SMA

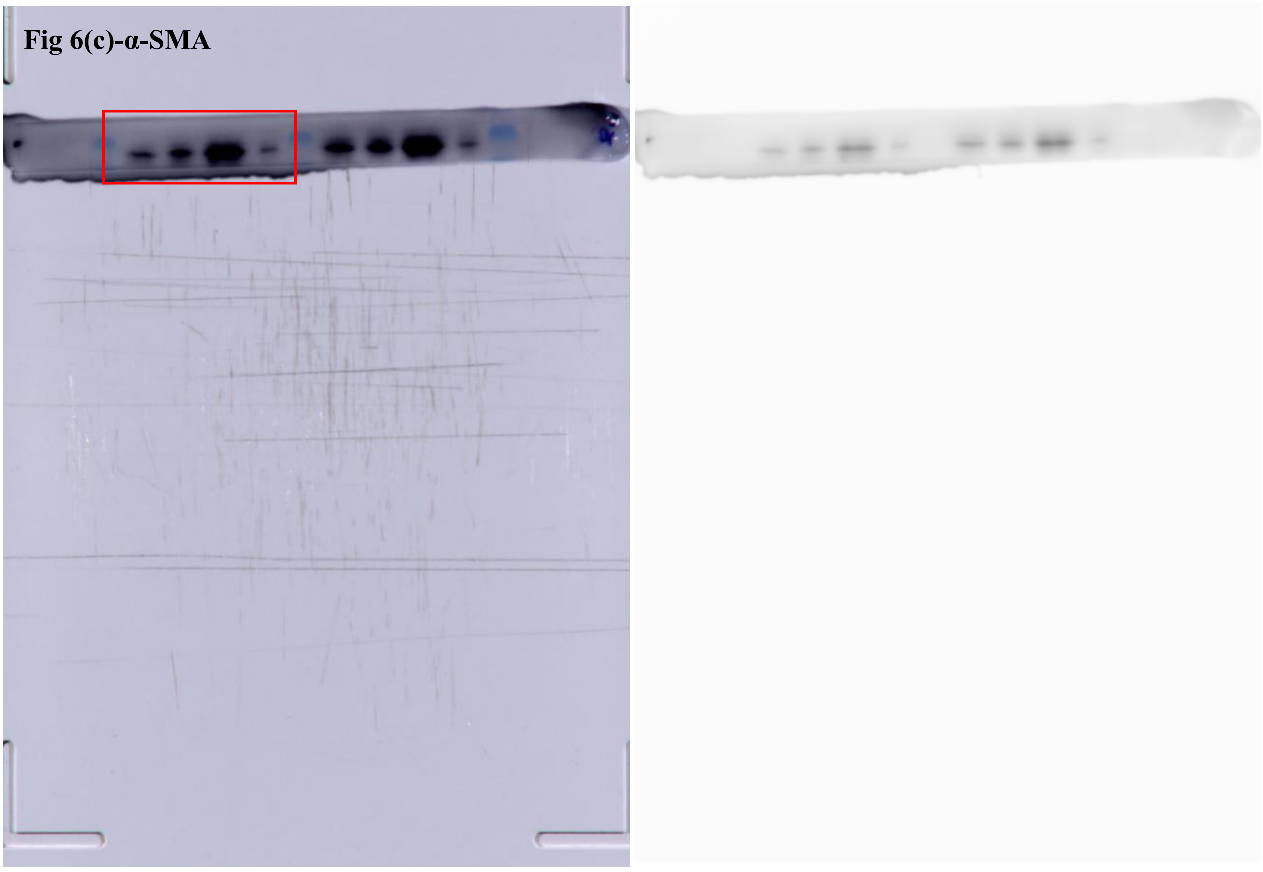

Fig 6(c)-FAP

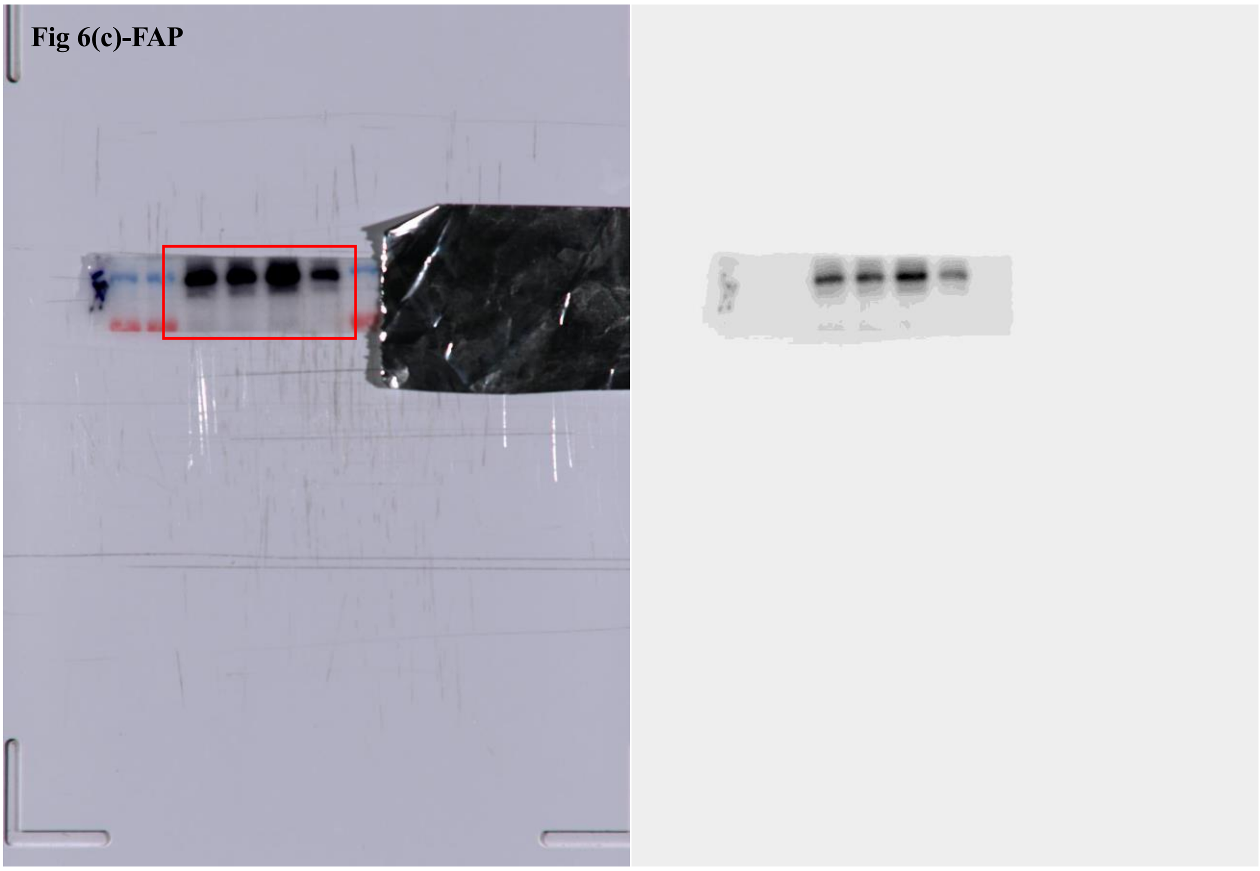

All replicates for WB images in the article

Fig 1(c)-GAPDH

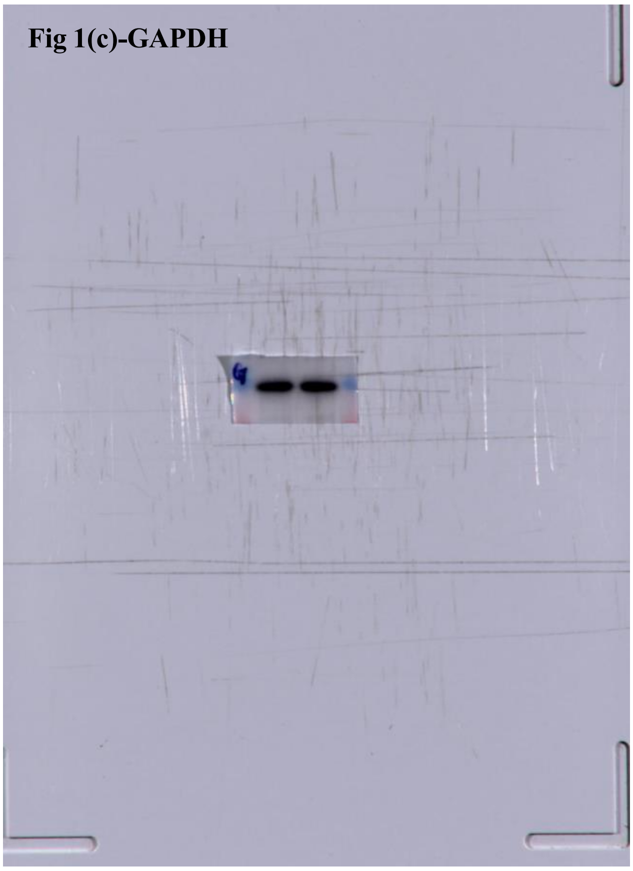

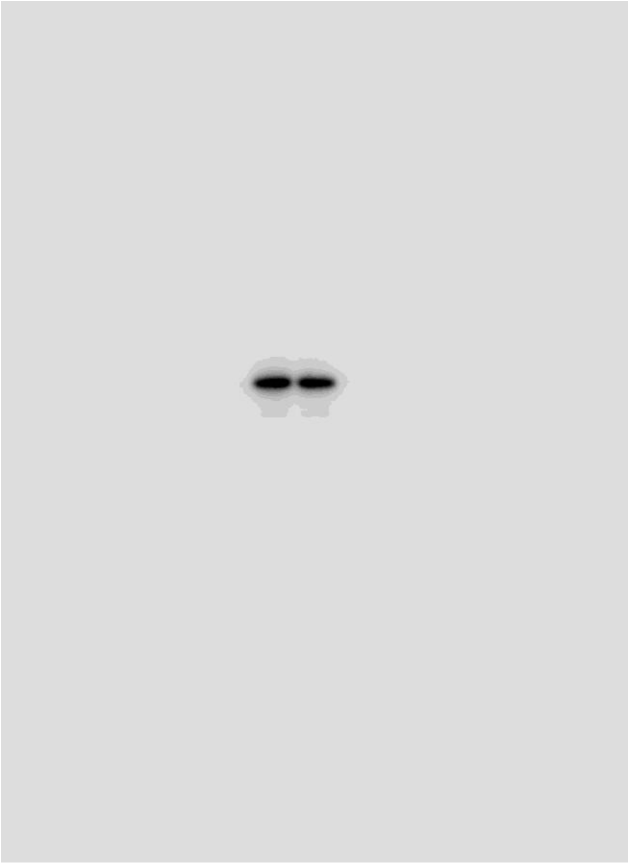

Fig 1(c)-α-SMA

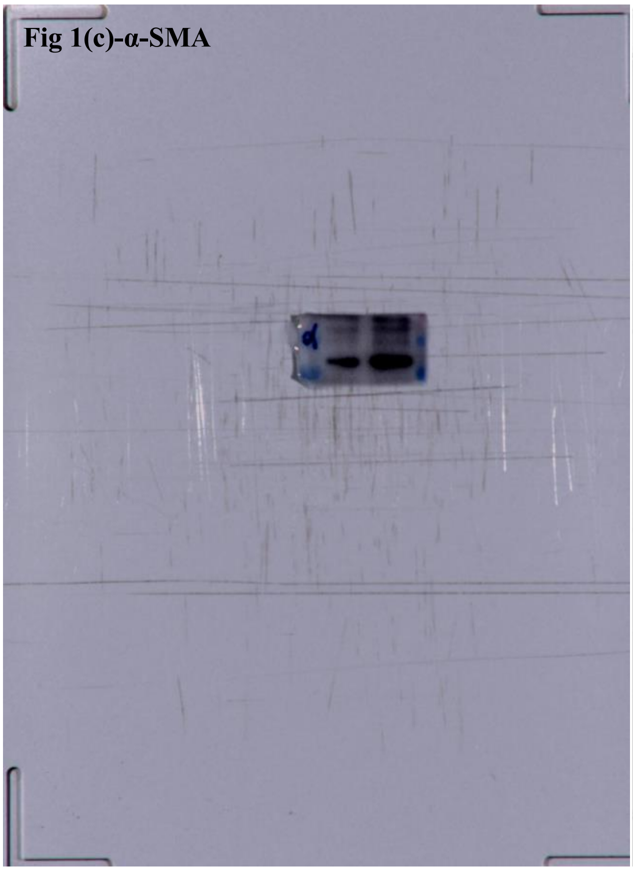

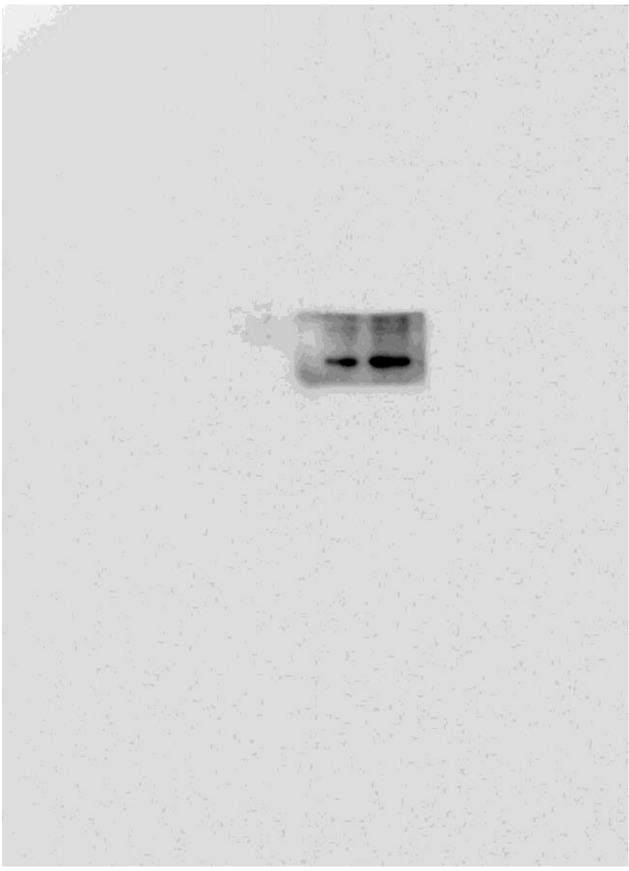

Fig 1(c)-FAP

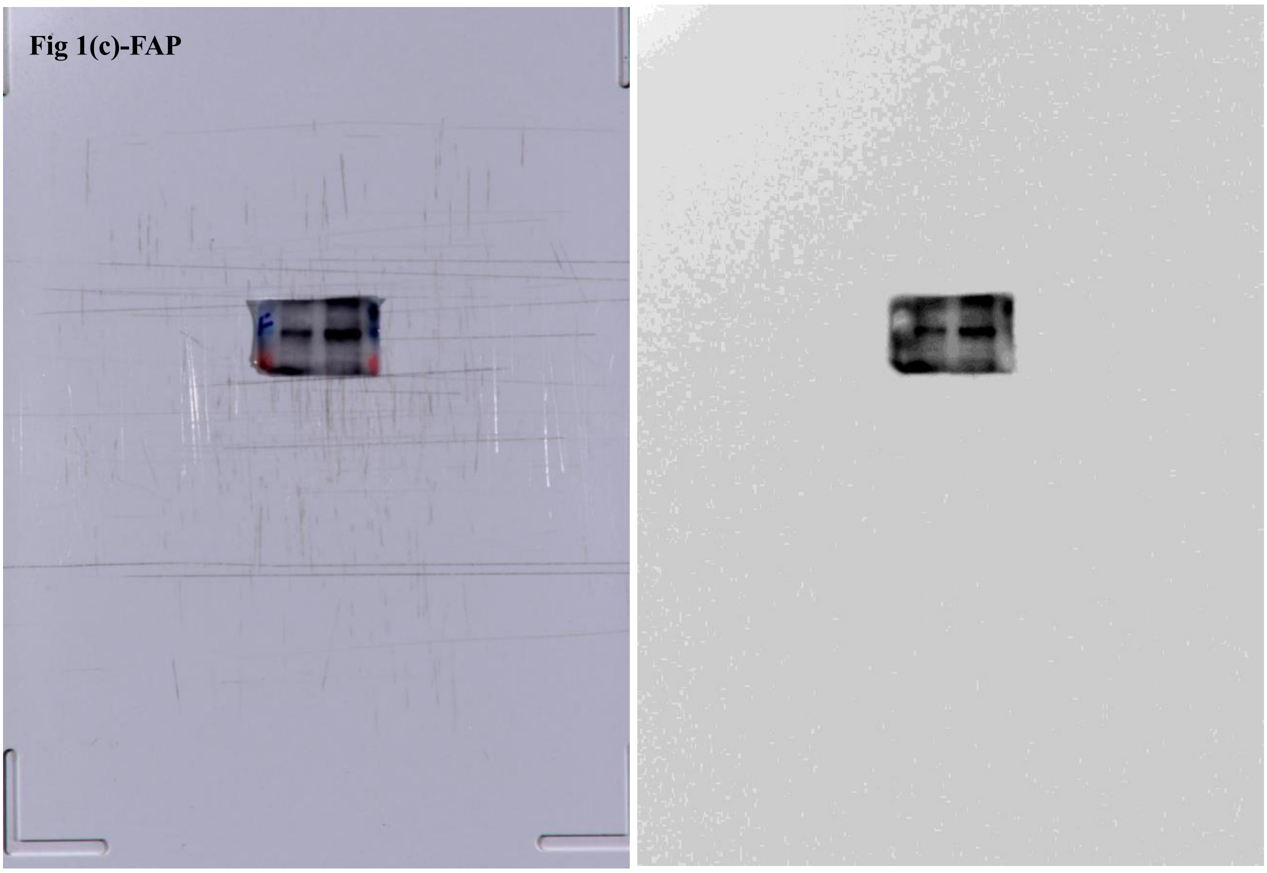

Fig 1(c)-GAPDH

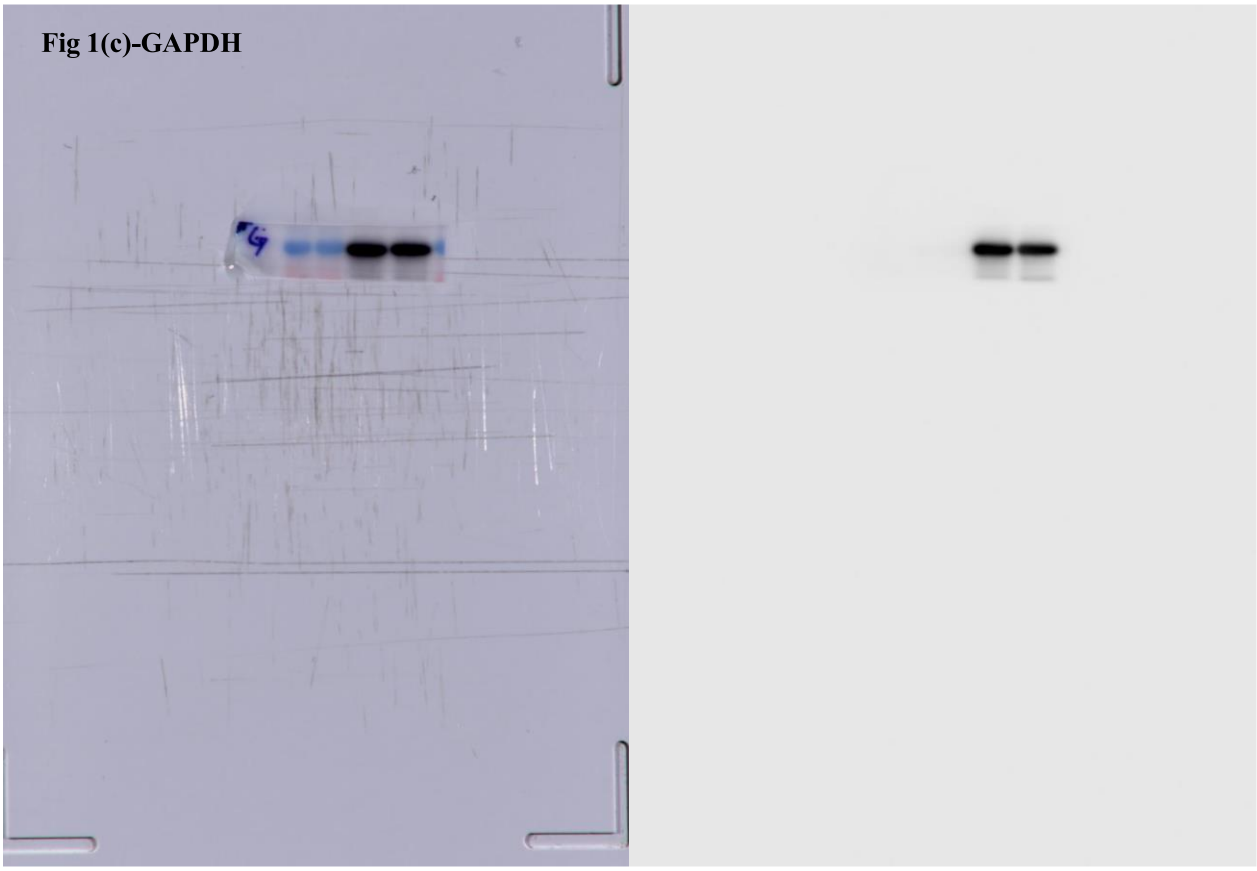

Fig 1(c)-α-SMA

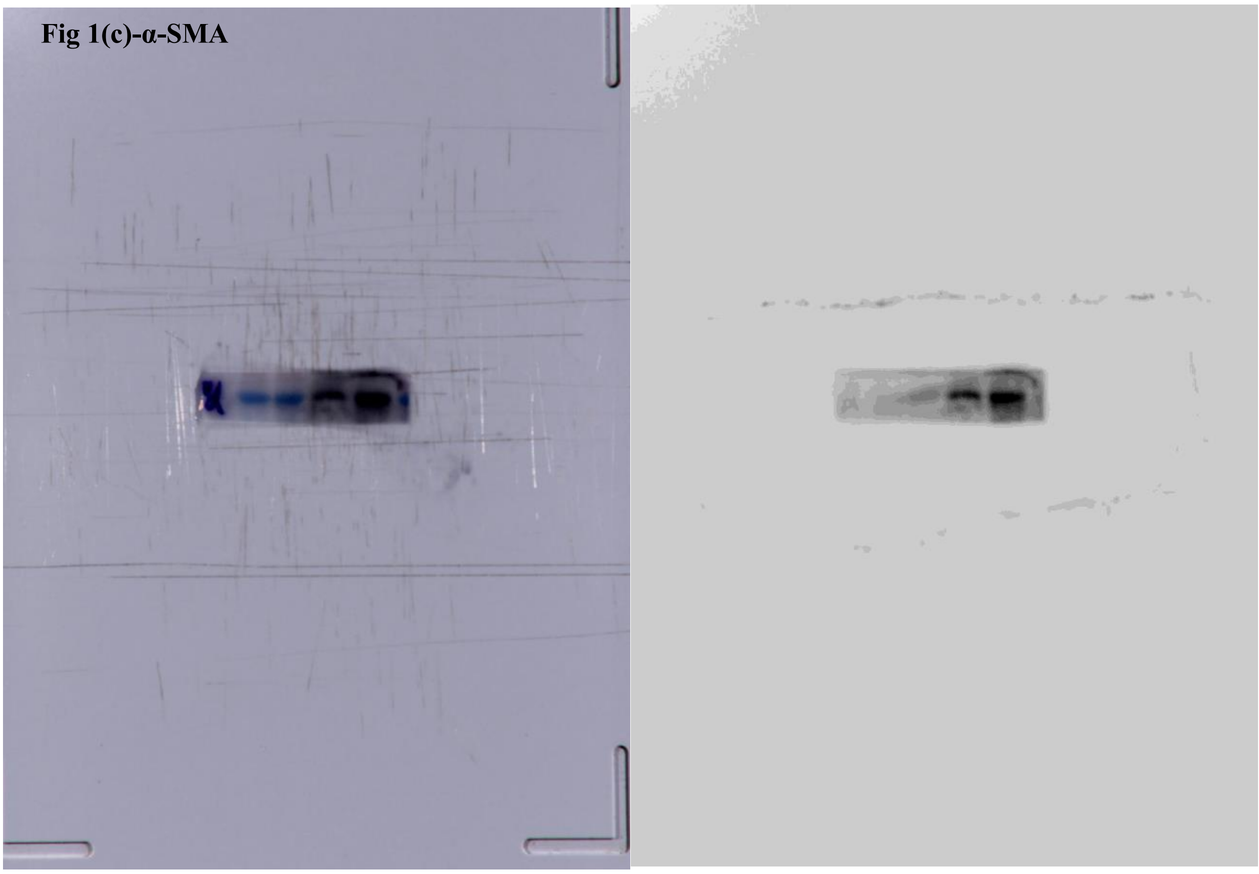

Fig 1(c)-FAP

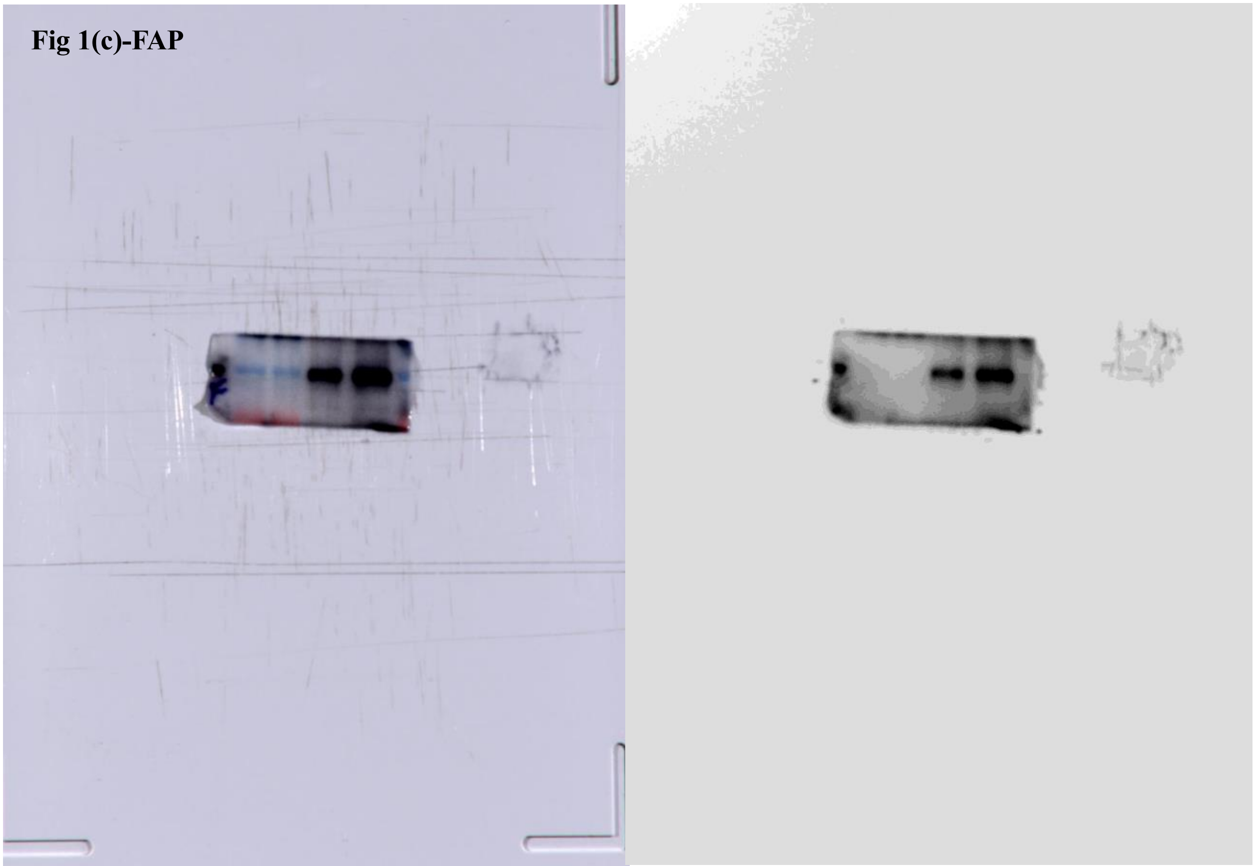

Fig 1(c)-GAPDH

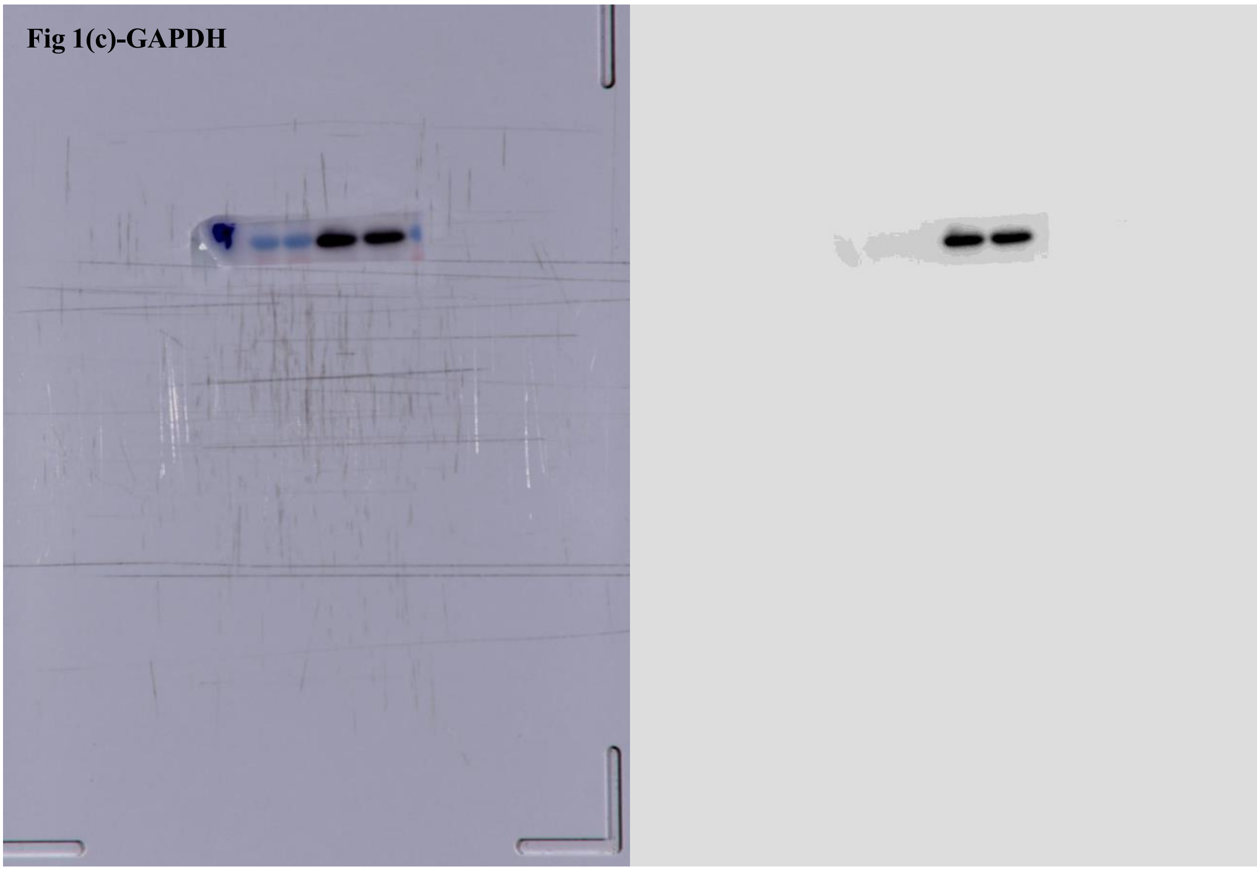

Fig 1(c)-α-SMA

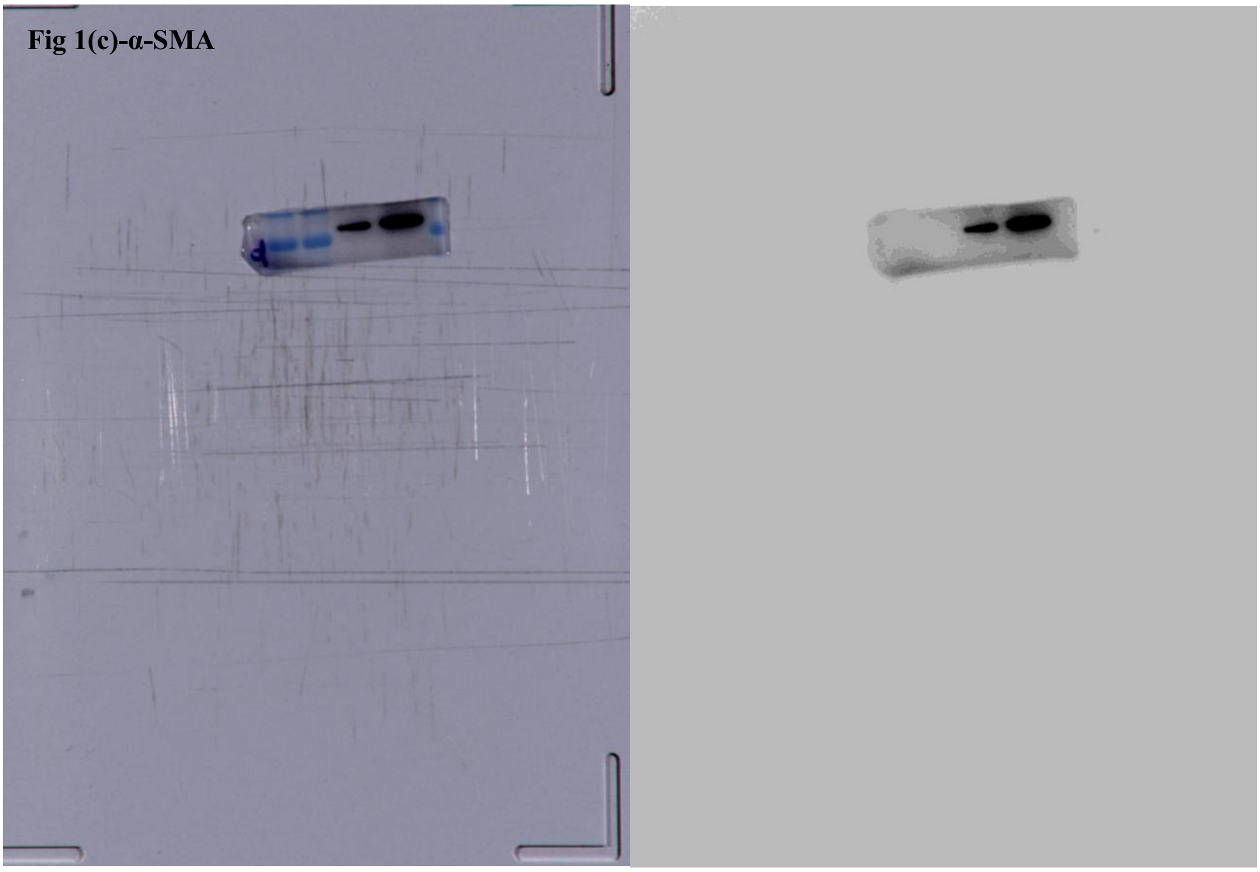

Fig 1(c)-FAP

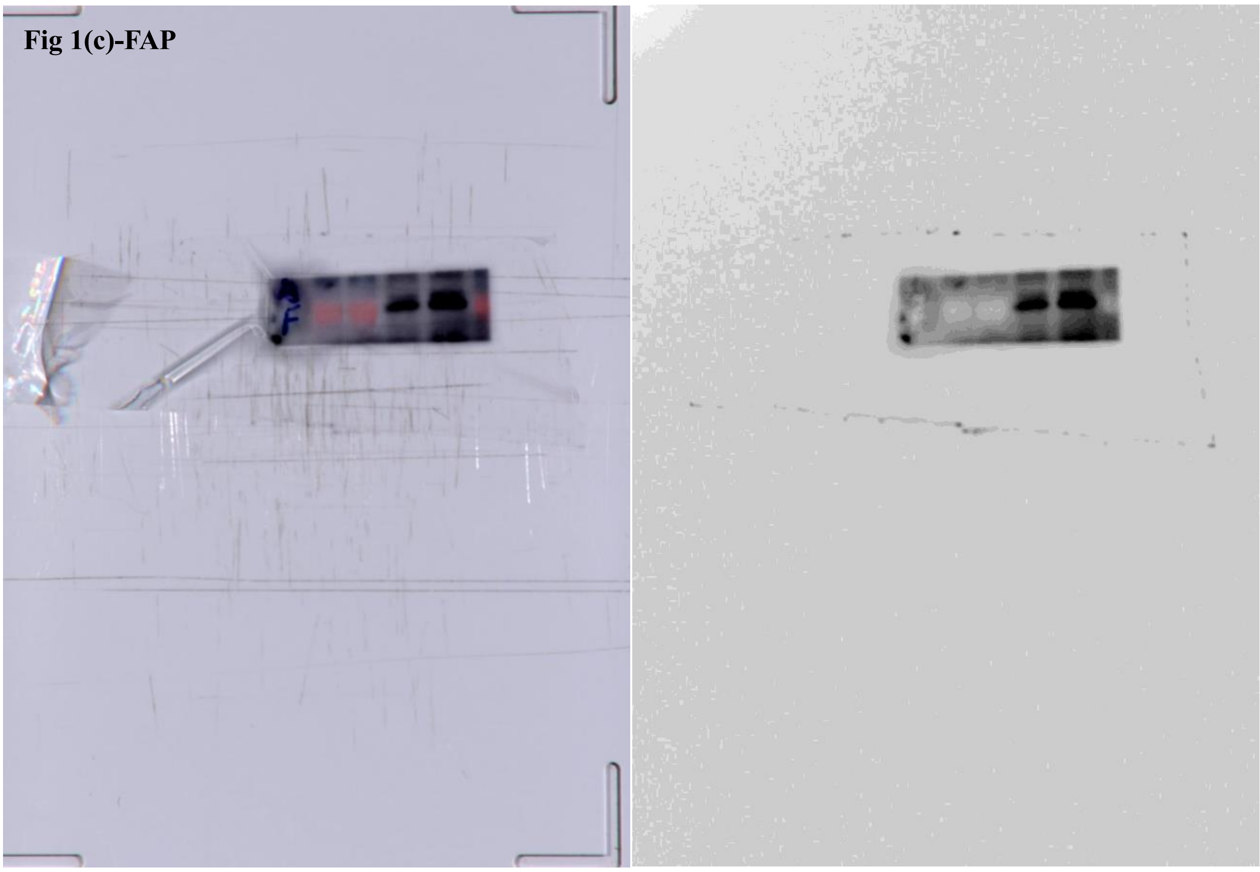

Fig 2(c)-CD63

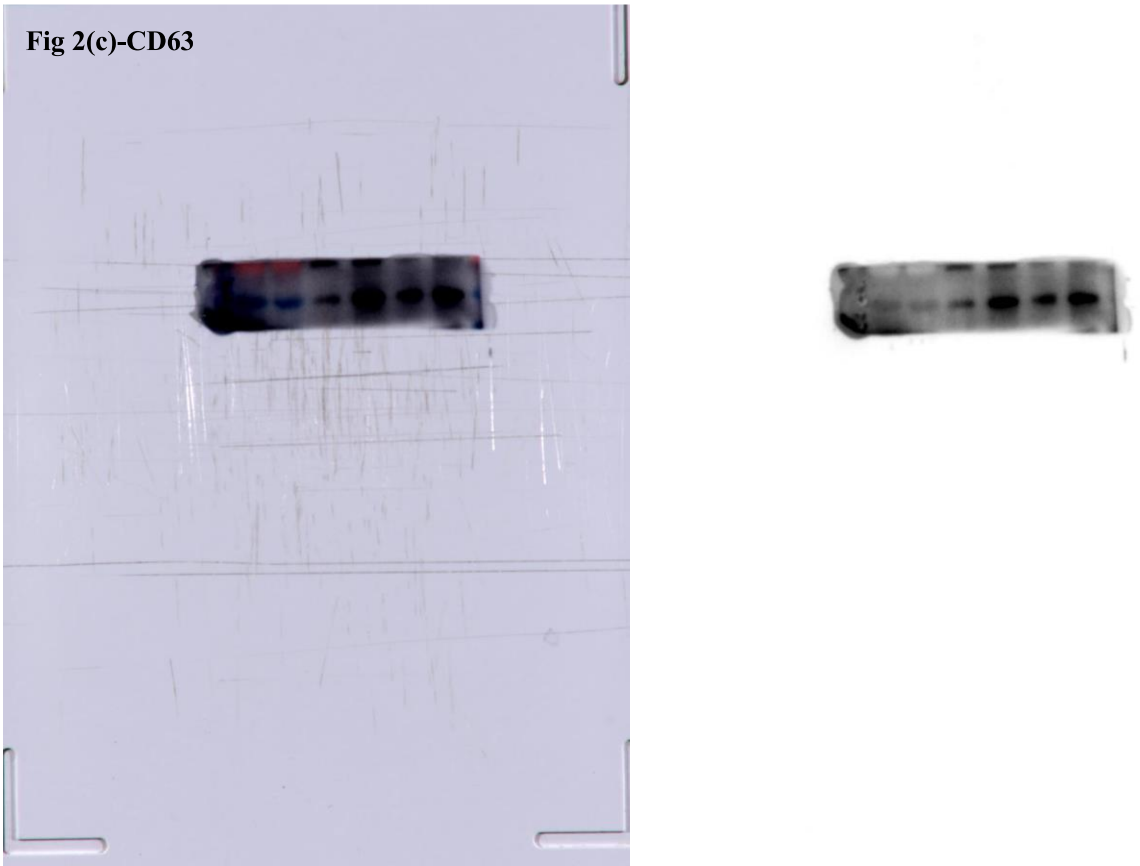

Fig 2(c)-CD81

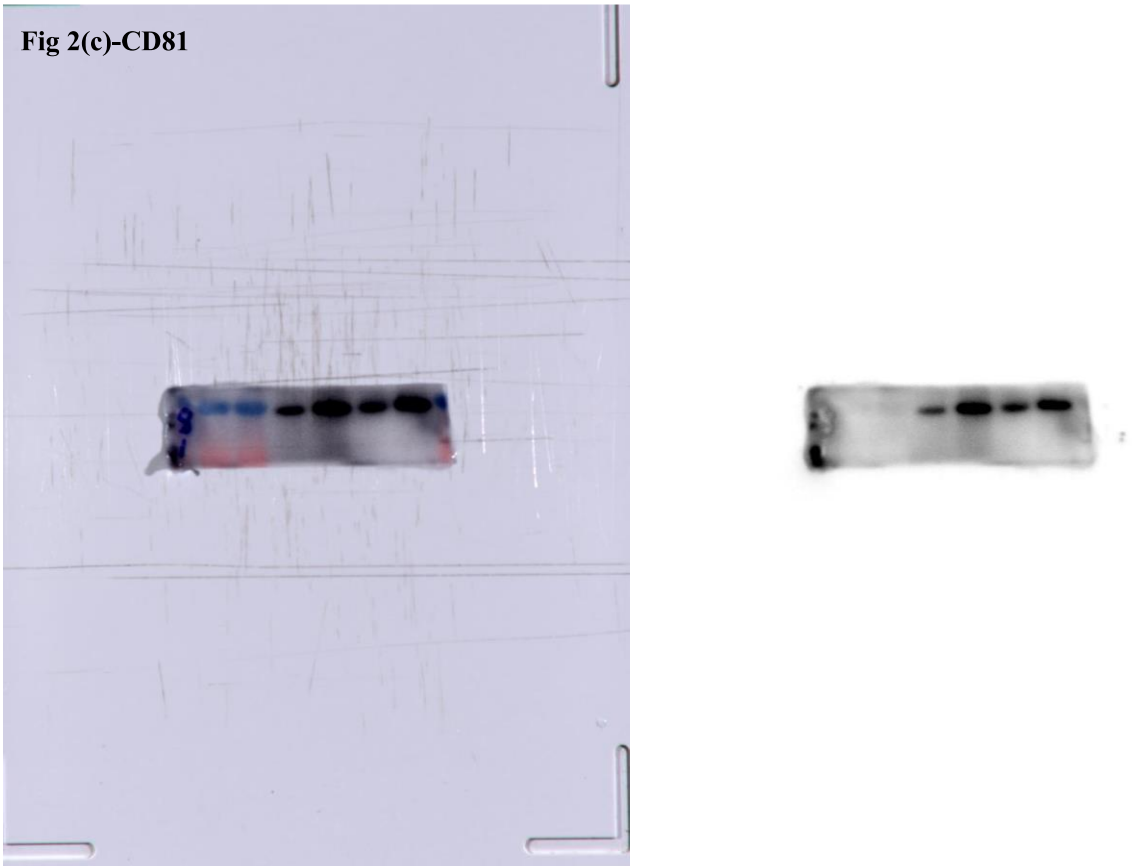

Fig 2(c)-CD63

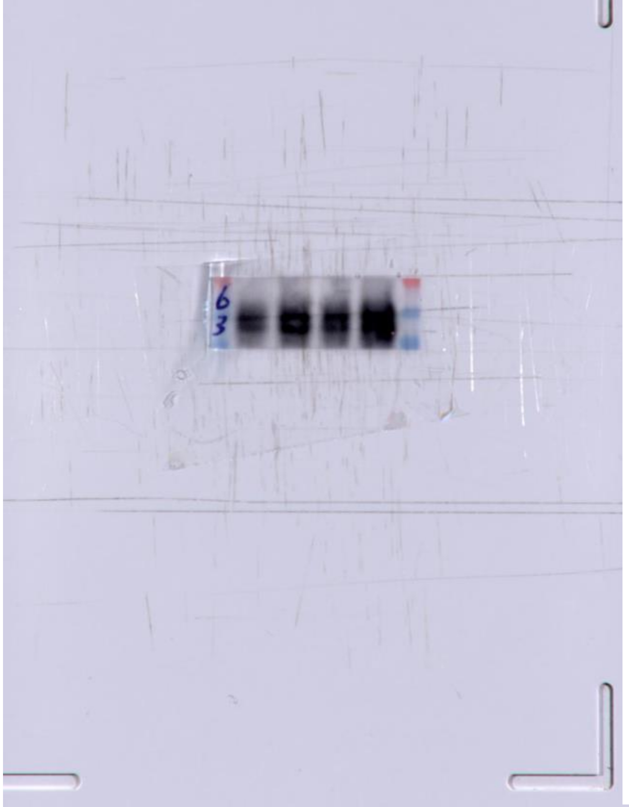

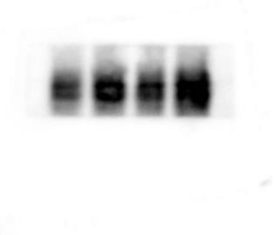

Fig 2(c)-CD81

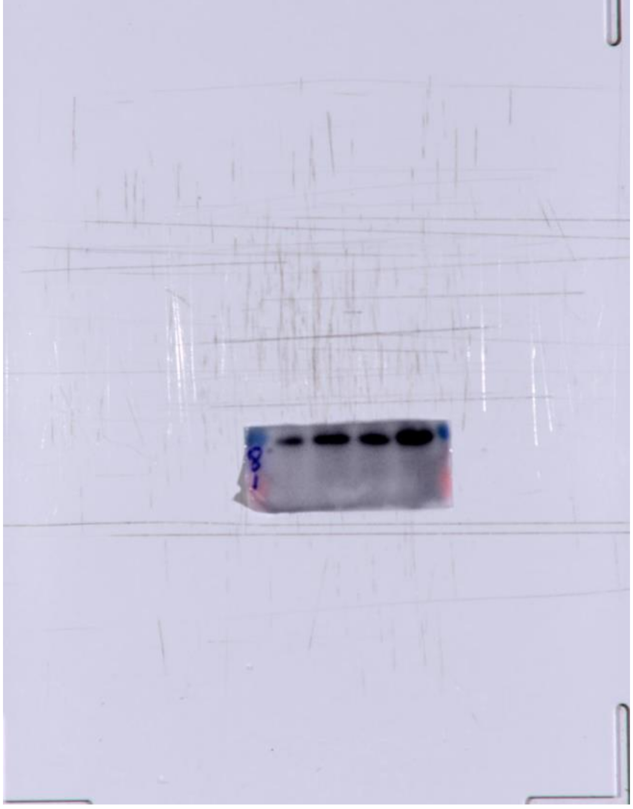

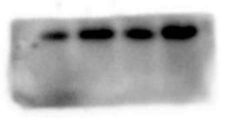

Fig 2(c)-CD63

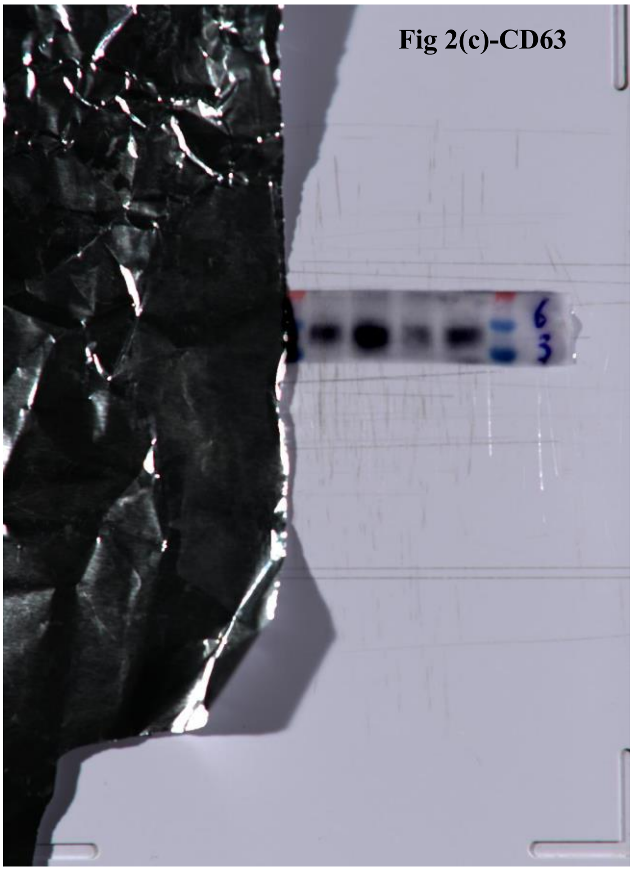

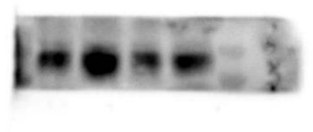

Fig 2(c)-CD81

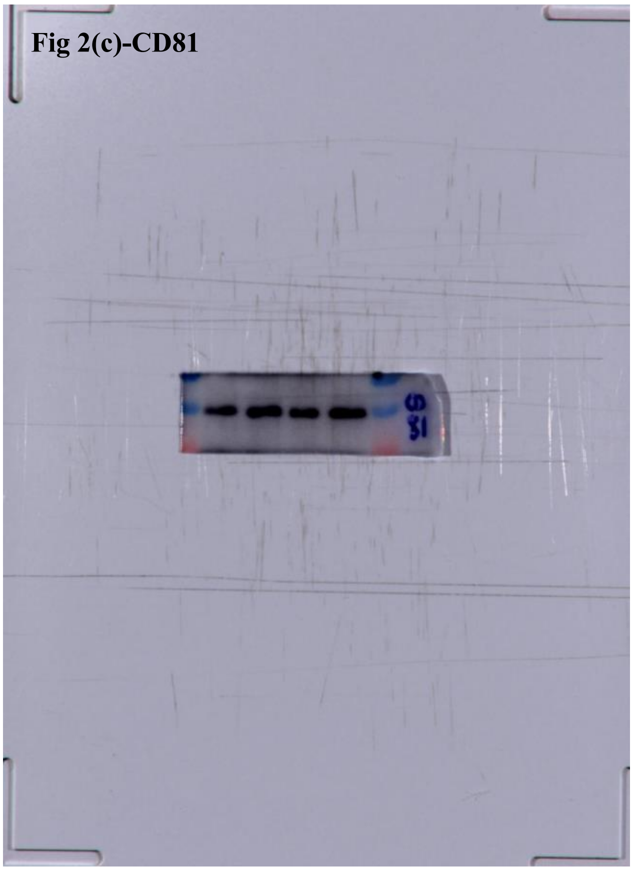

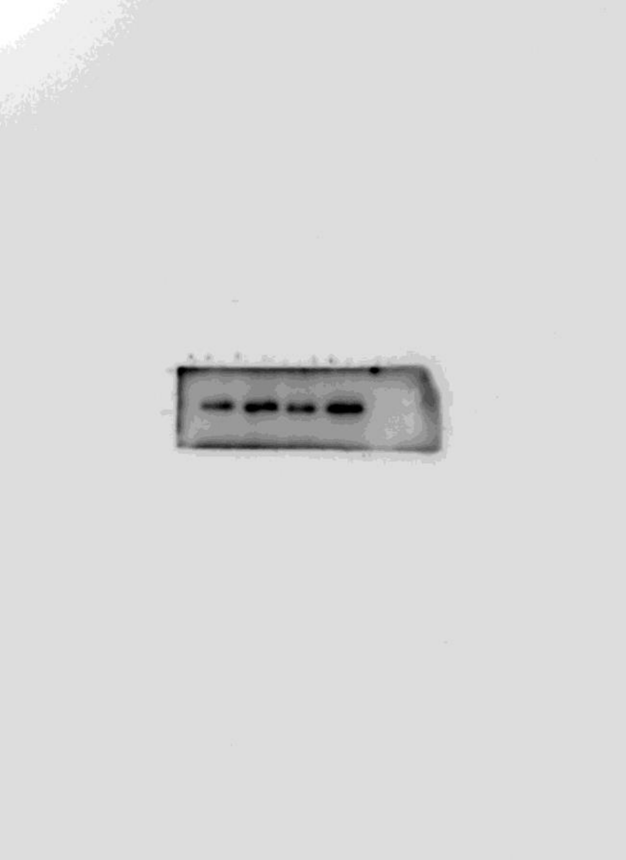

Fig 6(c)-GAPDH

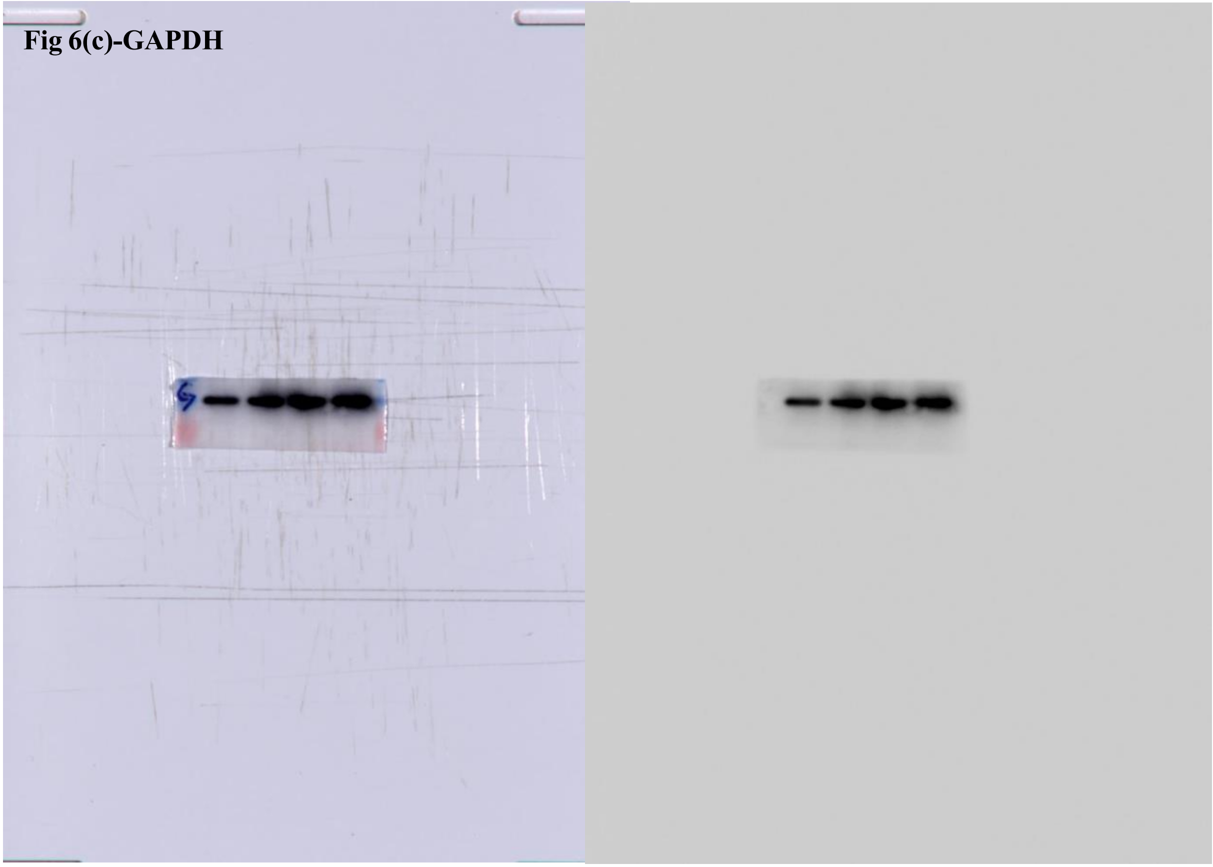

Fig 6(c)-α-SMA

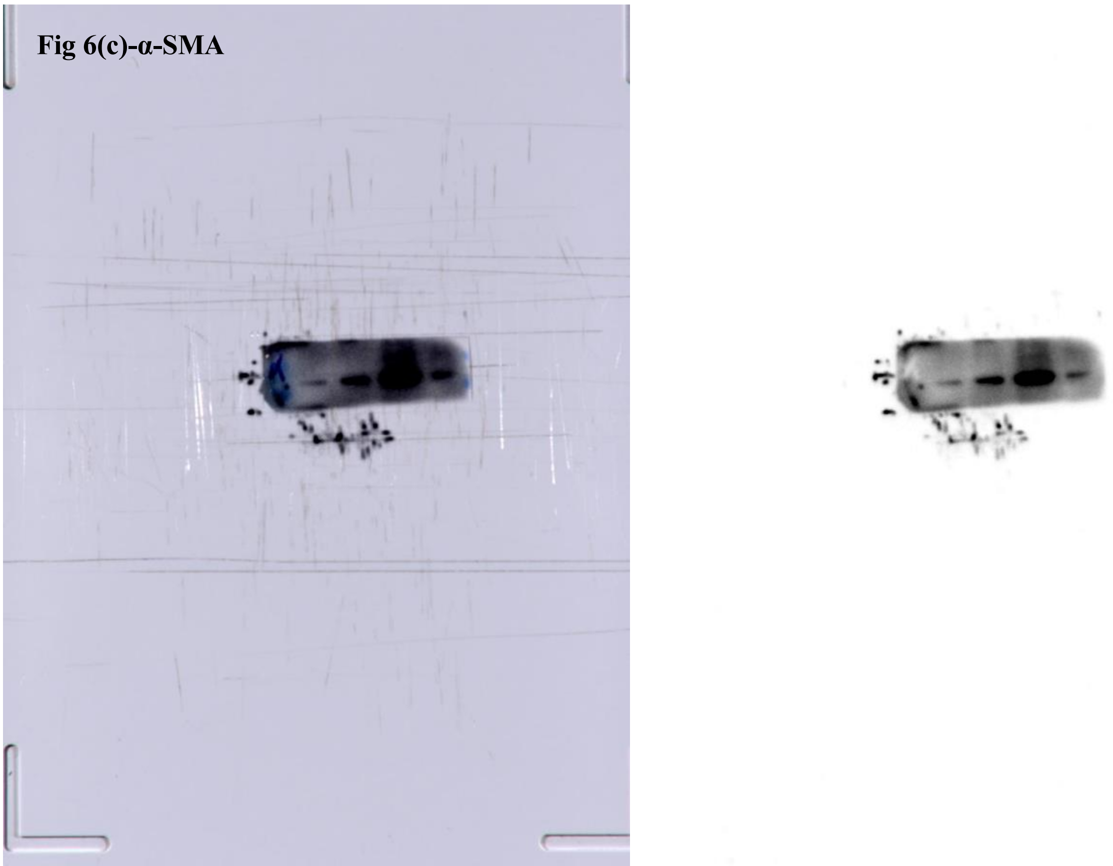

Fig 6(c)-FAP

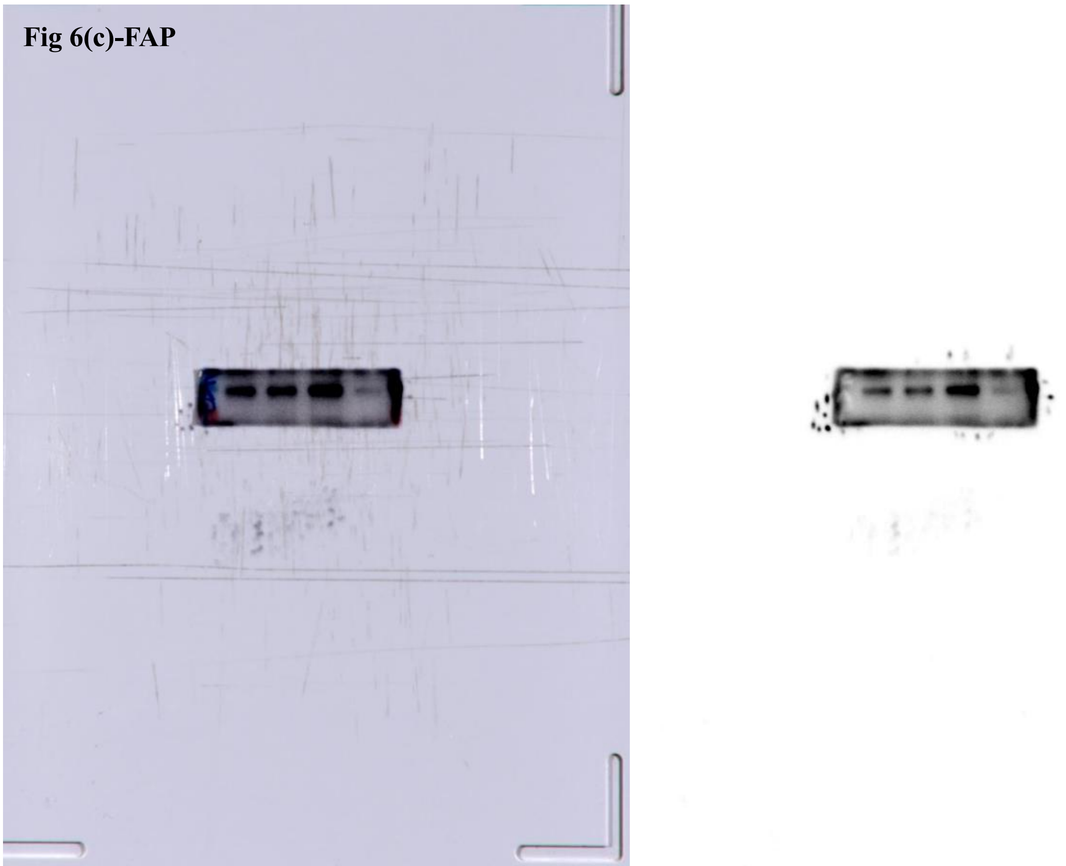

Fig 6(c)-GAPDH

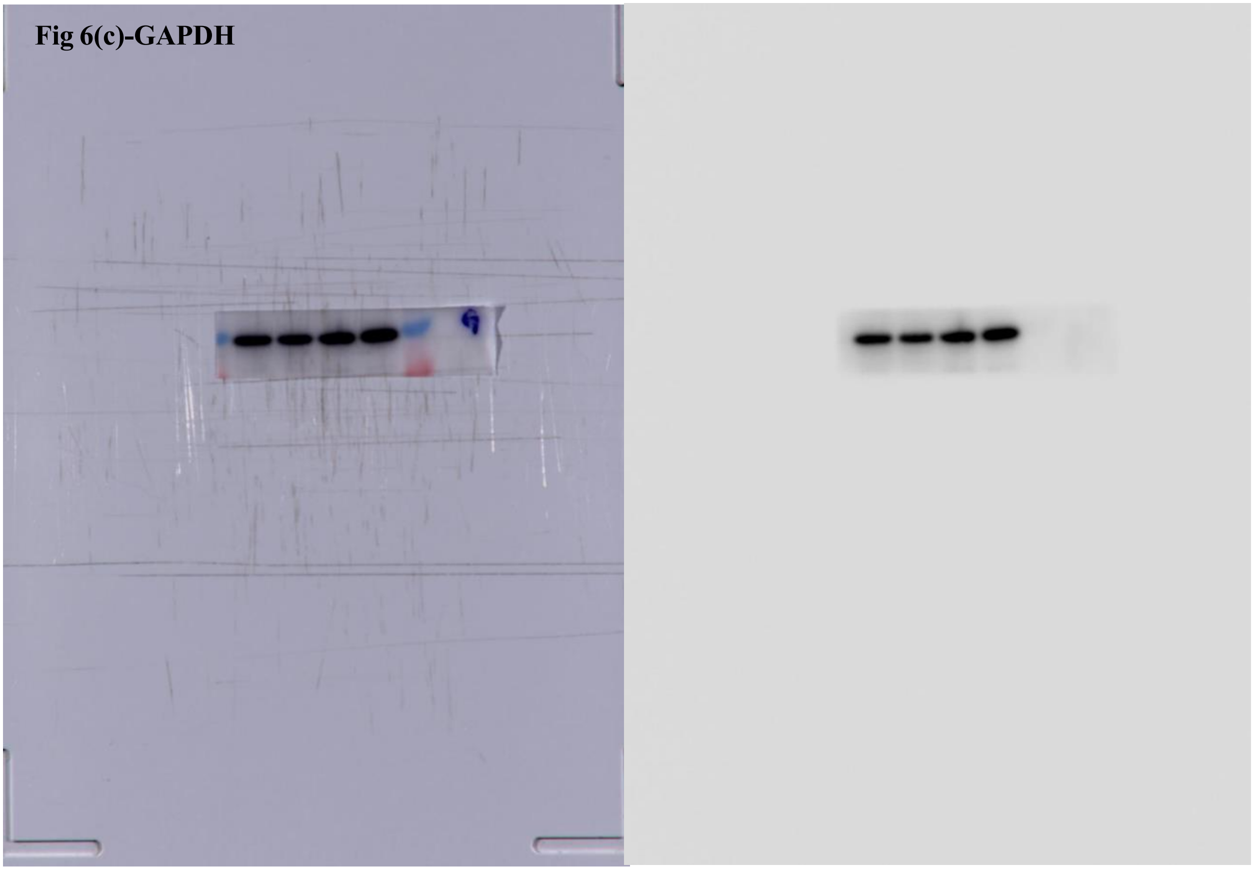

Fig 6(c)-α-SMA

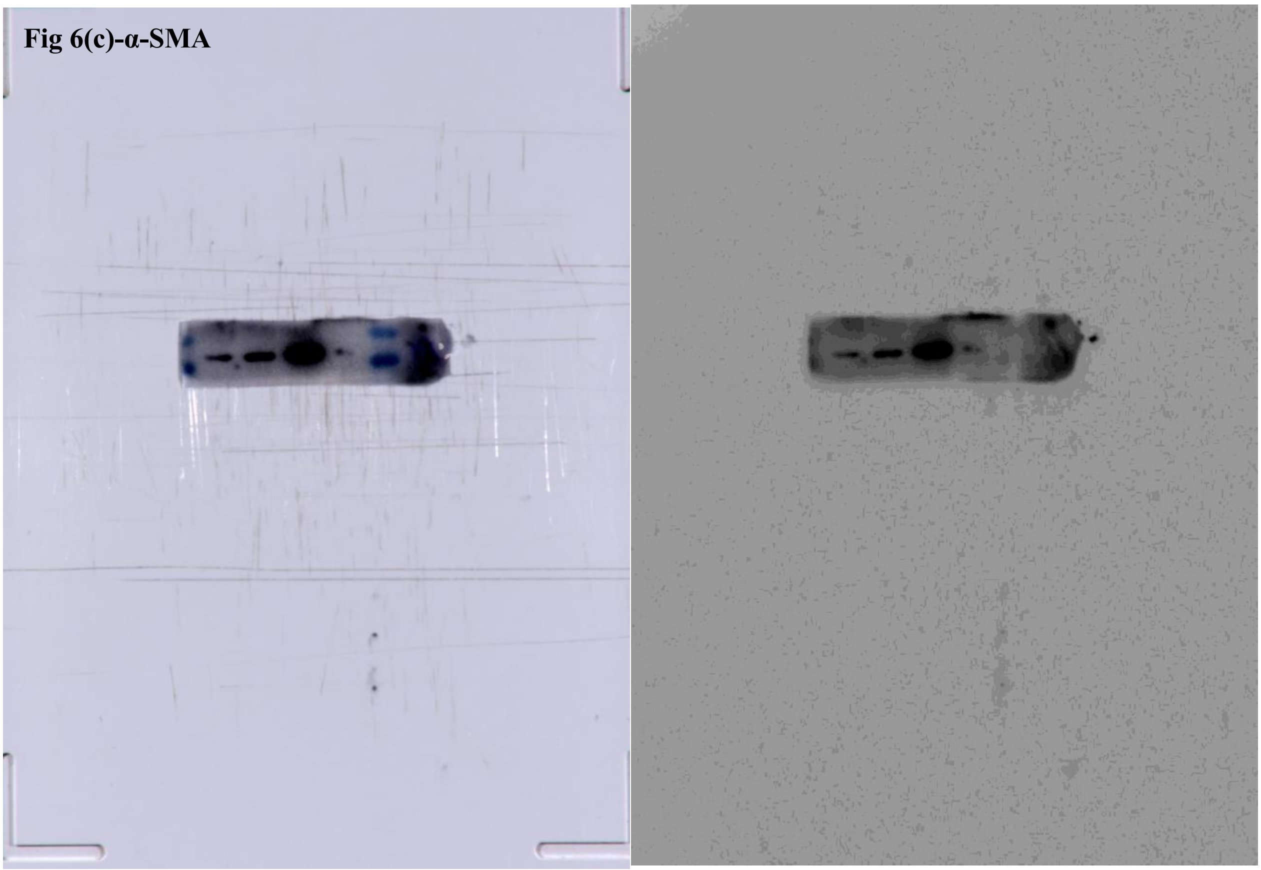

Fig 6(c)-FAP

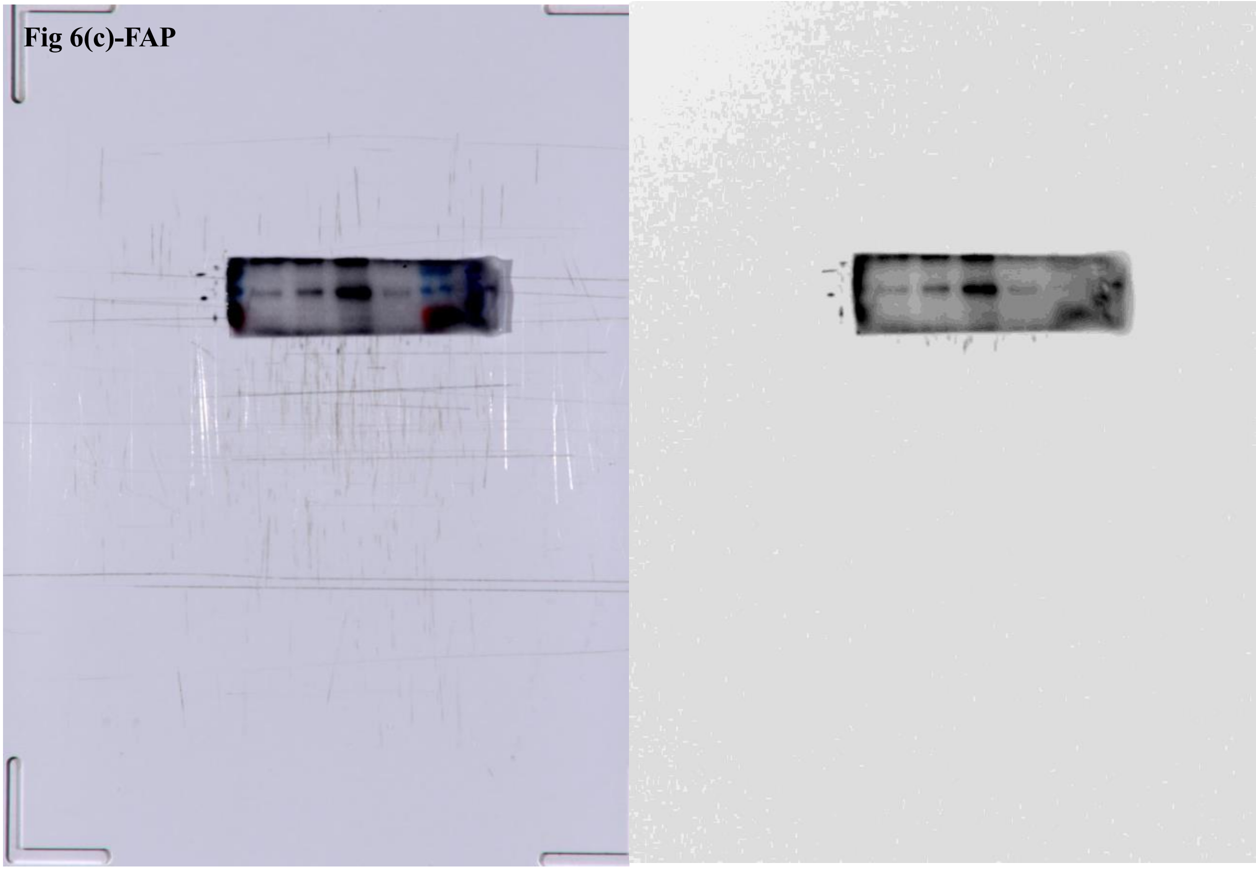

Fig 6(c)-GAPDH

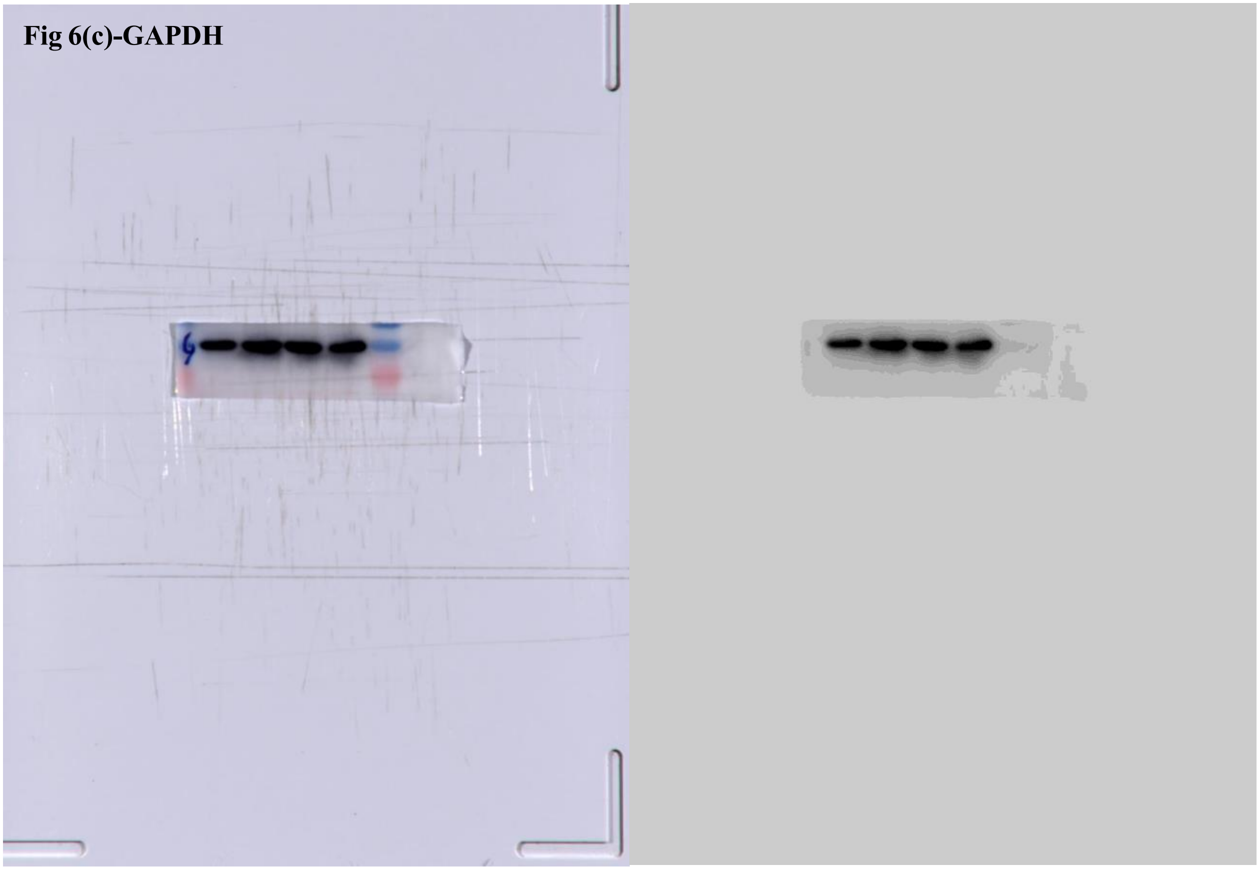

Fig 6(c)-α-SMA

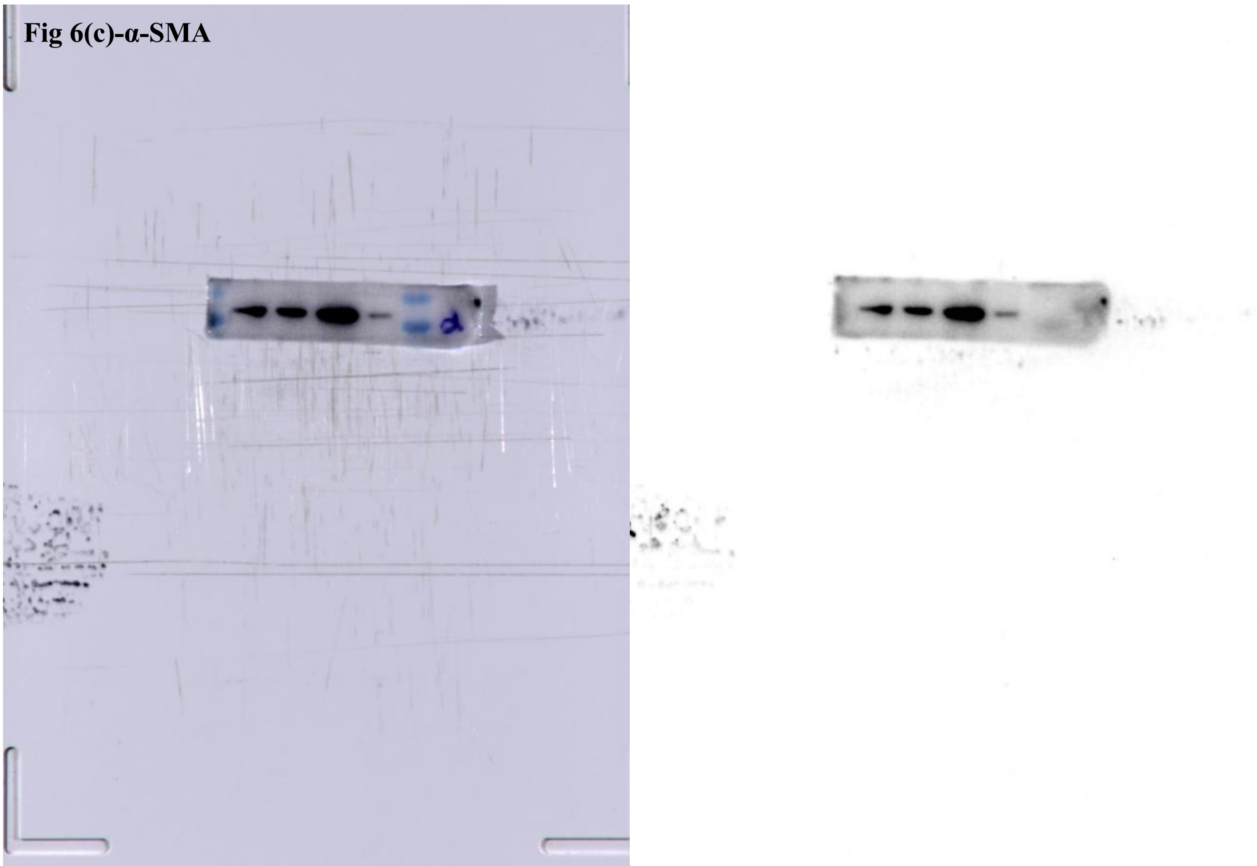

Fig 6(c)-FAP

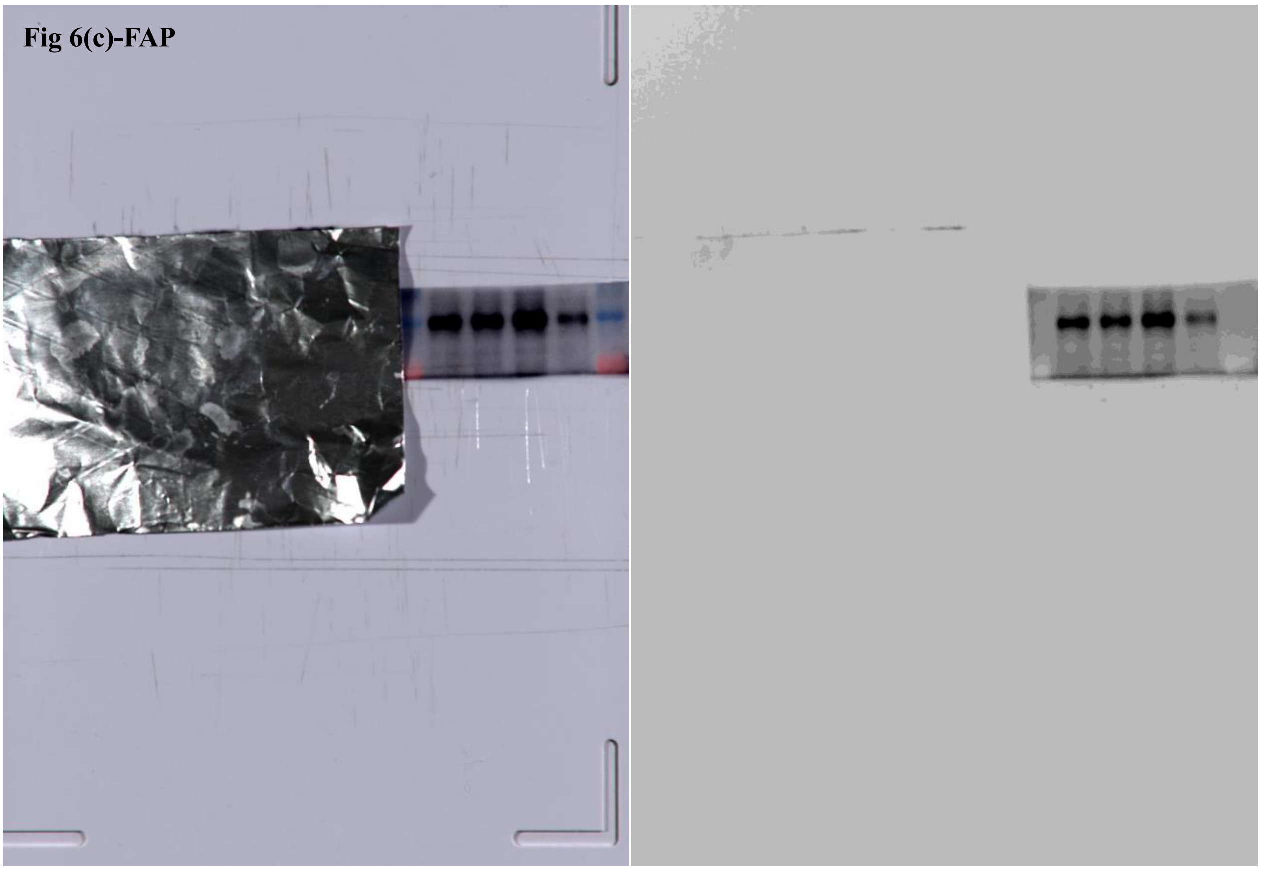

Supplement: Supplementary file 1 — Supplementary Information. [file 41598_2023_36092_MOESM1_ESM.pdf]
